# Distinctive Gait Variations and Neuroimaging Correlates in Alzheimer's Disease and Cerebral Small Vessel Disease

**DOI:** 10.1002/jcsm.13616

**Published:** 2024-11-17

**Authors:** Xia Zhou, Wen‐Wen Yin, Chao‐Juan Huang, Si‐Lu Sun, Zhi‐Wei Li, Ming‐Xu Li, Meng‐Meng Ren, Ya‐Ting Tang, Jia‐Bin Yin, Wen‐Hui Zheng, Chao Zhang, Yu Song, Ke Wan, Yue Sun, Xiao‐Qun Zhu, Zhong‐Wu Sun

**Affiliations:** ^1^ Department of Neurology The First Affiliated Hospital of Anhui Medical University Hefei China; ^2^ Department of Rehabilitation Medicine The First Affiliated Hospital of Anhui Medical University Hefei China; ^3^ Department of Neurology The First Affiliated Hospital of USTC Hefei China; ^4^ Department of Radiology The First Affiliated Hospital of Anhui Medical University Hefei China; ^5^ Department of Neurology The Second Affiliated Hospital of Anhui Medical University Hefei China

**Keywords:** Alzheimer's disease, cerebral small vessel disease, cognition, gait

## Abstract

**Background:**

Both Alzheimer's disease (AD) and cerebral small vessel disease (CSVD) manifest in cognitive impairment and gait disorders. The precise similarities and differences in gait characteristics and underlying neuroimaging mechanisms remain unclear.

**Methods:**

A total of 399 participants were enrolled: 132 with probable ad, including 98 with mild cognitive impairment due to ad (ad‐MCI) and 34 with ad dementia, and 185 with CSVD and 82 healthy controls. CSVD patients with cognitive impairment, including subcortical vascular mild cognitive impairment (svMCI) and subcortical vascular dementia, were grouped as subcortical vascular cognitive impairment (SVCI). Voxel‐based morphology analysis assessed grey matter volume (GMV), while cerebral blood flow (CBF) was derived from 3D‐arterial spin labelling data. Gait metrics included the timed up and go (TUG) test, dual‐task TUG (DTUG) test, Berg balance scale (BBS), dual‐task cost (DTC), step length, gait speed, cadence and coefficient of variation of gait. The relationships among structural and perfusion variations, gait metrics and cognitive function were examined.

**Results:**

SVCI patients exhibited greater gait impairments and variability than those with ad, while ad patients experienced higher DTC (*p <* 0.05). These differences were most evident in the MCI stage. In ad, gait speed correlated with GMV in the left middle occipital gyrus (*F* = 6.149), middle temporal gyrus (*F* = 4.595), right precuneus (*F* = 5.174) and other regions (all *p <* 0.025). In SVCI, gait speed was linked to thalamic GMV (*F* = 6.004, *p <* 0.025). Altered CBF in the parietal lobe and precuneus was associated with DTUG (*F* = 5.672), gait speed (*F* = 4.347) and BBS (*F* = 4.153) in ad, while cerebellar CBF related to TUG (*F* = 6.042), DTUG (*F* = 4.857) and BBS (*F* = 7.097) in SVCI (all *p <* 0.025). In ad‐MCI, memory mediated the effect of hippocampal volume on DTC (indirect effect: −2.432, 95% CI [−5.503, −0.438]), while executive function (indirect effect: −2.920, 95% CI [−7.227, −0.695]) and processing speed (indirect effect: −2.286, 95% CI [−5.174, −0.484]) mediated the effect on DTUG. In svMCI, executive function mediated the effect of thalamic volume on step length (indirect effect: 2.309, 95% CI [0.486, 4.685]) and gait speed (indirect effect: 2.029, 95% CI [0.142, 4.588]), while processing speed mediated the effect on step length (indirect effect: 1.777, 95% CI [0.311, 4.021]).

**Conclusions:**

Different gait disorder characteristics and mechanisms were observed in ad and CSVD patients. In ad, gait is associated with volume/perfusion in posterior brain regions, whereas in SVCI, it relates to thalamic volume and cerebellar perfusion. Cognitive impairment mediates the effect of hippocampal and thalamic volumes on gait in ad‐MCI and svMCI, respectively.

## 1 Background

Cognitive and motor impairments become increasingly prevalent with aging, posing a significant public health concern and significantly impacting the quality of life for older individuals [1]. These symptoms are closely interconnected, with cognition playing a key regulatory role in gait and balance, and poor gait performance predicting dementia incidence several years in advance [2]. Alzheimer's disease (ad) is the most common cause of degenerative dementia, accounting for 60%–80% of all cases. Gait dysfunction is reported in over 30% of individuals with the ad precursor, amnestic mild cognitive impairment (aMCI) [3]. Vascular dementia (VD), the second most common type of dementia after ad, has garnered widespread concern owing to its high occurrence in older individuals. Cerebral small vessel disease (CSVD) is the prevalent cause of vascular cognitive impairment, presenting white matter hyperintensities (WMHs), lacune, cerebral microbleeds, perivascular spaces or cerebral atrophy on magnetic resonance imaging (MRI) [4]. Gait disturbance is a significant symptom of CSVD, second only to cognitive impairment, characterized by an insidious onset, gradual progression, a substantial increase in the risk of falls and a lack of helpful treatment in the late stage. Despite motor symptoms prevalence in ad and CSVD, their underlying neural mechanisms remain insufficiently explored, particularly in understanding the distinctions in characteristics and mechanisms of gait disorders between ad and CSVD.

Given the potential for improving treatment outcomes and preventive value, gait problems have attracted considerable attention in the predementia stages, yet their underlying mechanisms remain unclear. Previous studies primarily focused on comparing gait in individuals with aMCI to those with non‐amnestic mild cognitive impairment (non‐aMCI) but yielded inconsistent results [3, 5, 6]. For instance, Allali et al. observed greater variability in stride length and width during usual pace walking in non‐aMCI compared to aMCI [5]. In contrast, other studies reported the opposite pattern [3, 6]. These differences may be attributed to the heterogeneity of non‐aMCI, encompassing vascular mild cognitive impairment (MCI) due to large or small vessel lesions, Parkinson's disease (PD)‐MCI or other degenerative or VD subtypes.

Atherosclerotic CSVD constitutes a relatively homogeneous group of cerebrovascular disorders affecting small vessels or microvasculature, resulting in subcortical cognitive impairment (SVCI), including subcortical vascular MCI (svMCI) and subcortical VD (sVD). Clinically, CSVD frequently overlaps with ad, posing challenges in diagnosis and complicating the differentiation of gait‐related issues [7]. Previously, studies have focused on unravelling the neural mechanism behind gait disorder in ad and CSVD, primarily by investigating isolated brain structural or perfusion variations, given their respective significance as degenerative and cerebrovascular diseases. Research indicates that slower gait speed in individuals with MCI and ad correlates with smaller grey matter volume (GMV) in the medial temporal lobe and motor brain regions [8]. Koblinsky et al. observed that gait speed was directly related to thalamic cerebral blood flow (CBF) in older adults, and their sensitivity analysis revealed that WMHs accounted for a 3% decrease in variance and a 4% decrease in the observed effect size between them [9]. In the early stages of ad, increased postural sway was associated with reduced CBF in the cortex, as measured by single‐photon emission computed tomography [10]. No study has systematically compared distinctive gait differences across different stages of cognitive impairment within and between ad and CSVD nor has there been an exploration of the relationship between brain structure/perfusion variations and gait in both conditions. Furthermore, most study samples comprised older Western adults, and variations in the anatomical distribution of CSVD between Eastern and Western populations have been reported [11]. Limited research has been conducted in Asian populations, and a significant gap exists in identifying biomarkers to differentiate gait problems associated with cognitive impairment in ad and CSVD.

Therefore, the objectives of our study are as follows: first, to compare gait differences between ad and CSVD and to assess gait variations among patients with different levels of cognitive impairment and healthy controls (HC) within each condition; second, to examine the relationship between gait characteristics, GMV and CBF in AD and CSVD patients, particularly at the early MCI stage. This study may help identify subtle differences in gait disturbances linked to CSVD and AD and detect early gait impairments through imaging markers.

## 2 Methods

### 2.1 Participants

This study employed an observational cross‐sectional design. We recruited 399 participants from the First Affiliated Hospital of Anhui Medical University, including 132 individuals with probable ad—98 with MCI due to ad (ad‐MCI) and 34 with ad dementia (ADD); 185 individuals with CSVD—76 with subcortical vascular non‐cognitive impairment (sNCI), 78 with svMCI and 31 with sVD; and 82 HC. The CSVD patients experiencing cognitive impairment were termed as the SVCI group, including svMCI and sVD. Diagnostic criteria were based on previous studies and guidelines [12, 13, 14]. The inclusion and exclusion criteria for ad, CSVD and HC are detailed in the supplementary materials and Figure S1. All participants provided written informed consent, and the research ethics committee of the First Affiliated Hospital of Anhui Medical University approved this study (reference number: Quick‐PJ 2023‐14‐76).

### 2.2 Clinical and Neuropsychological Assessments

Demographic information was collected from all participants. Trained neuropsychological technicians performed neuropsychological tests for all participants, completing each patient's testing within a day. Global cognitive functions were assessed using the Mini‐mental state examination (MMSE) and Montreal cognitive assessment (MoCA). Memory was evaluated using the auditory verbal learning test (AVLT); executive functions were measured with the trail making test‐B (TMT‐B) and Stroop colour‐word test‐C (SCWT‐C); attention was tested using the digit span test (DST); visuospatial abilities were examined using the clock drawing task (CDT); and processing speed was gauged using TMT‐A, SCWT‐A and SCWT‐B. Clinical dementia rating (CDR) was used to evaluate the extent of cognitive impairment. Raw scores for each test were standardized using *z‐* transformation based on the mean and standard deviation of the control group. Each domain score was calculated by averaging the *z* scores from relevant neuropsychological tests. For time‐consuming tests like SCWT and TMT, *z* scores were inverted to (−z), because higher scores indicated worse performance.

### 2.3 Mobility

Gait assessment included qualitative, semiquantitative and quantitative evaluations. The qualitative analysis involved clinical assessments, observing gait patterns and identifying muscle or joint issues. The semiquantitative assessment used clinical scales like the timed up and go (TUG) test, dual‐task TUG (DTUG) test and Berg balance scale (BBS) for rating. In the DTUG test, participants identified numbers containing the digit seven while walking (e.g., 7, 17 and 27) and recorded metrics included time taken and number of errors. The dual‐task cost (DTC) was calculated as (DTUG − TUG)/TUG. Quantitative gait assessment utilized the intelligent device for energy expenditure and activity (IDEEA), a portable instrument known for its high test–retest reliability and validity in recording and analysing gait data [15]. IDEEA parameters, including step length, gait speed and cadence, were employed for the quantitative measurements. Variability was calculated as the coefficient of variation (CV): standard deviation/mean × 100. The *z* scores of gait parameters were used for further analysis.

### 2.4 MRI Acquisition and Processing

MRI data were acquired using a 3.0‐T MRI scanner (Discovery MR750w; GE, Milwaukee, WI) equipped with a 24‐channel head coil. The obtained sequences included 3D‐T1, pseudocontinuous arterial spin labelling (PCASL), T2‐FLAIR and susceptibility‐weighted imaging (SWI). The Supporting Information displays the detailed sequence parameters and preprocessing steps for 3D‐T1 and PCASL.

### 2.5 Voxel‐Based Morphometry (VBM) Analysis and CBF Analysis

VBM analysis was conducted using the computational anatomy toolbox 12 (CAT12) (http://www.neuro.uni‐jena.de/cat/) with statistical parametric mapping software 12 (SPM12, https://www.fil.ion.ucl.ac.uk/spm). Software (AW Server GE Healthcare) automatically generated CBF maps. The downloaded CBF data were analysed using SPM8 (https://www.fil.ion.ucl.ac.uk/spm/software/spm8/). The Supporting Information (Method) displays details of the VBM and CBF analysis.

### 2.6 Statistical Analyses

Statistical analysis was conducted using IBM SPSS Statistics 23.0 and R‐4.2.2. Continuous variables are presented as mean (standard deviation) and analysed using one‐way variance analysis for three‐group comparisons. Categorical variables, expressed as *n* (%), were compared using the chi‐square test. The Bonferroni correction was employed to compare gait parameters across groups, controlling for age, gender, body mass index (BMI) and hypertension (corrected *p <* 0.05). SPM software was utilized to assess correlations between gait and whole‐brain GMV or CBF, controlling for age, gender, BMI and/or total intracranial volume (TIV), with family‐wise error (FWE) correction applied (corrected *p <* 0.05). Detailed methods for the multiple comparisons of the results are provided in Table S1. Partial correlation and mediation analysis were performed to explore the relationships among brain structure/perfusion variations, gait and cognition, with age, gender, education, BMI and/or TIV as covariates. PROCESS macro (https://www. processmacro.org/) software was used for mediation analysis. Based on 5000 bootstrap realizations, a significant indirect effect was determined if the bootstrap 95% confidence interval (CI) excluded zero. The statistical significance threshold was set at 0.05.

## 3 Results

### 3.1 Demographic and Clinical Characteristics

Table 1 illustrates significant differences in age, gender, education and BMI among the groups. Patients in the ad and CSVD groups were older than those in the HC group, with the ad group having a higher proportion of females. Additionally, the ad group exhibited lower education levels and BMI compared to the CSVD and HC groups. Notably, the prevalence of hypertension significantly differed among the groups. Concerning cognitive function comparison, both the ad and CSVD groups demonstrated impairment in overall cognitive function, as evidenced by lower MMSE and MoCA scores and deficits in executive/attention functions (SCWT, DST and TMT tests) compared to the HC group. Memory, attention, executive function, language, visuospatial function and information processing were compromised in both the ad and CSVD groups.

**TABLE 1:** Demographics data of all participants.

	HC (*N* = 82)	AD (*N* = 132)	CSVD (*N* = 185)	*p* value
Age, years	59.54 (7.09)	62.65 (7.44)^#^	66.32 (8.01)^#^	< 0.001^a^
Gender, female	46 (56%)	82 (62%)	89 (48%)^†^	0.045^b^
Education, years	9.43 (3.96)	7.79 (3.55)^#^	8.10 (5.23)	0.028^a^
BMI, kg/m^2^	23.49 (2.34)	22.86 (2.64)	23.70 (3.24)^†^	0.035^a^
Alcohol abuse, *n* (%)	18 (22%)	37 (28%)	45 (24%)	0.578^b^
Current smoking, *n* (%)	14 (17%)	28 (21%)	51 (28%)	0.136^b^
Hypertension, *n* (%)	21 (26%)	47 (36%)	97 (52%)^#^ ^,^ ^†^	< 0.001^b^
Diabetes mellitus, *n* (%)	4 (5%)	15 (11%)	27 (14%)^#^	0.072^b^
Dyslipidaemia, *n* (%)	13 (16%)	22 (17%)	40 (22%)	0.402^b^
CAD, *n* (%)	4 (5%)	6 (5%)	15 (8%)	0.374^b^
Global cognition				
MMSE	28.60 (1.26)	22.68 (5.67)^#^	24.09 (5.86)^#^	< 0.001^a^
MoCA	25.70 (2.43)	16.90 (5.66)^#^	19.26 (6.70)^#^ ^,^ ^†^	< 0.001^a^
Memory				
AVLT‐IR	17.98 (3.87)	11.22 (5.08)^#^	12.76 (5.52)^#^ ^,^ ^†^	< 0.001^a^
AVLT‐SR	6.15 (2.00)	3.12 (2.46)^#^	3.85 (2.93)^#^ ^,^ ^†^	< 0.001^a^
AVLT‐LR	5.88 (2.16)	2.74 (2.51)^#^	3.49 (2.91)^#^ ^,^ ^†^	< 0.001^a^
AVLT‐recognition	21.44 (2.32)	17.96 (3.67)^#^	18.45 (3.93)^#^	< 0.001^a^
Attention				
DS‐forward	13.07 (2.89)	10.96 (3.45)^#^	11.46 (3.73)^#^	< 0.001^a^
DS‐backward	7.34 (2.56)	5.15 (2.31)^#^	5.41 (2.57)^#^	< 0.001^a^
Executive				
TMT‐B	110.60 (52.62)	135.54 (56.62)^#^	153.87 (70.63)^#^ ^,^ ^†^	< 0.001^a^
SCWT‐C	34.86 (10.04)	49.25 (28.43)^#^	46.72 (22.01)^#^	< 0.001^a^
Language				
Animal counts	18.46 (3.98)	13.02 (5.44)^#^	14.11 (5.21)^#^	< 0.001^a^
Visuospatial				
CDT	9.21 (1.28)	6.87 (2.65)^#^	7.34 (3.03)^#^	< 0.001^a^
Processing speed				
SCWT‐A	19.56 (5.62)	28.36 (15.35)^#^	29.19 (14.18)^#^	< 0.001^a^
SCWT‐B	22.22 (6.12)	37.26 (24.66)^#^	35.36 (17.87)^#^	< 0.001^a^
TMT‐A	60.70 (22.81)	93.60 (42.74)^#^	90.95 (41.80)^#^	< 0.001^a^
CDR	0 (0)	0.66 (0.32)^#^	0.43 (0.50)^#^ ^,^ ^†^	< 0.001
GDS	4.42 (4.51)	6.34 (4.97)^#^	5.78 (5.11)	0.022^a^
ADL	20 (0)	22.93 (5.81)^#^	22.64 (6.09)^#^	< 0.001^a^
TIV, mL	1469.73 (118.07)	1475.08 (134.67)	1516.17 (150.03)^#^ ^,^ ^†^	0.009^a^

*Note:* Data are given as *n* (%) and mean (SD).

Abbreviations: AD: Alzheimer's disease; adL: activities of daily living scale; AVLT‐IR: auditory verbal learning test immediate recall; AVLT‐LR: auditory verbal learning test long‐term recall; AVLT‐SR: auditory verbal learning test short‐term recall; BMI: body mass index; Cad: coronary artery disease; CDR: clinical dementia rating; CDT: clock drawing test; CSVD: cerebral small vessel disease; DS: digital span test; GDS, geriatric depression scale; HC: healthy control; MMSE: Mini‐Mental State Examination; MoCA: Montreal Cognitive Assessment; SCWT: Stroop colour‐word test; TIV: total intracranial volume; TMT: trail making test.

^a^ One‐way analysis of variance (ANOVA).

^b^ Chi‐square test.

^#^ Significant difference between the ad and CSVD groups compared to the HC group (Bonferroni correction, *p <* 0.05).

^†^ Significant difference between ad group and CSVD group (Bonferroni correction, *p <* 0.05).

### 3.2 The Comparison of the Gait Parameters Among Groups

**FIGURE 1:**
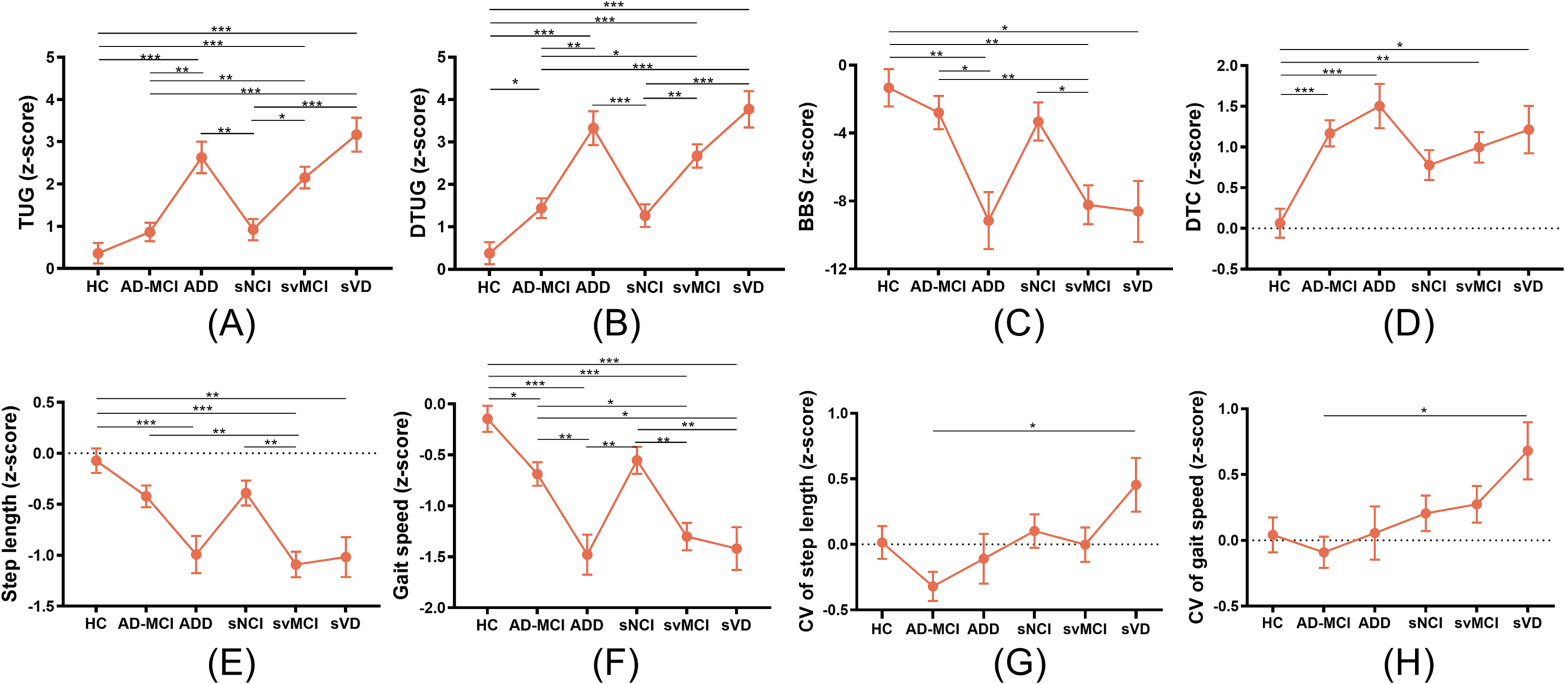
Variations in gait parameters of subgroups of ad (ad‐MCI + adD) and CSVD (sNCI + svMCI + sVD) versus HC, corrected for age, gender, BMI and hypertension (Bonferroni correction, *p <* 0.05). ad: Alzheimer's disease; adD (*N* = 34): ad dementia; ad‐MCI (*N* = 98): MCI due to probable ad; BBS: Berg balance scale; BMI: body mass index; CSVD: cerebral small vessel disease; CV: coefficient of variation; DTC: dual‐task cost; DTUG: dual‐task timed up and go test; HC (*N* = 82): healthy controls; sNCI (*N* = 76): subcortical vascular non‐cognitive impairment; sVD (*N* = 31): subcortical vascular dementia; svMCI (*N* = 78): subcortical vascular mild cognitive impairment; TUG: timed up and go test.

The ad‐MCI group demonstrated shorter time consumption in the TUG and DTUG than the svMCI group, with higher BBS scores. The ad‐MCI group also displayed a longer step length and faster gait speed than the svMCI group. A similar trend was observed when comparing adD and sVD groups. Regarding DTC, the adD group incurred the highest cost, slightly higher in the AD‐MCI group than in the svMCI group. The sVD group displayed the longest time consumption in the TUG and DTUG tests, along with the shortest step length and slowest gait speed. The sVD group also displayed the highest CV in gait speed and step length. In the comparison of CSVD subgroups, time consumption in the TUG and DTUG tests gradually increased (sNCI < svMCI < sVD). No significant difference was observed in gait metrics between the sNCI and HC groups (Figure 1, the raw data were presented in Table S4).

**FIGURE 2:**
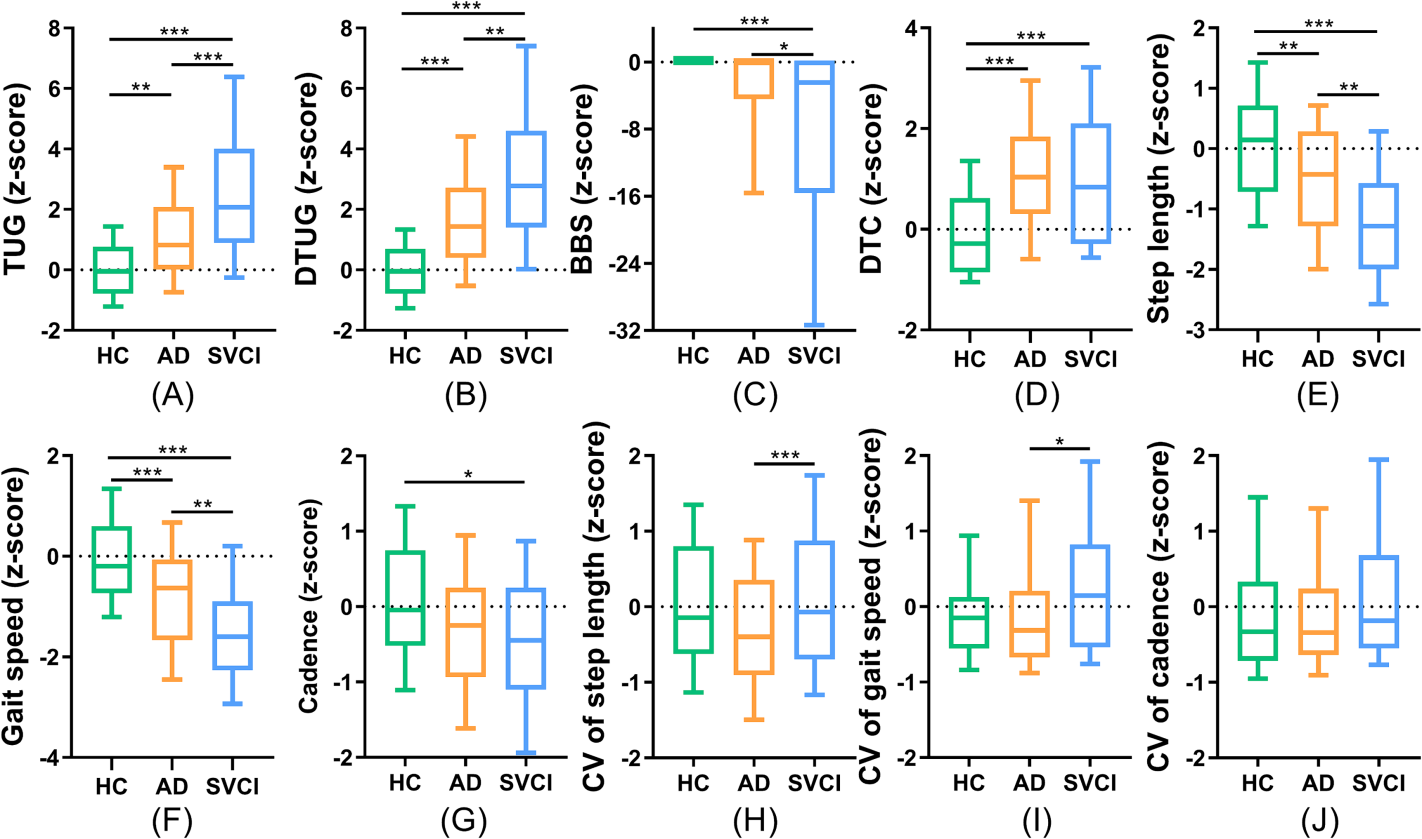
Difference of gait parameters in ad (ad‐MCI + ADD), SVCI (svMCI + sVD) and HC groups, corrected for age, gender, BMI and hypertension (Bonferroni correction, *p <* 0.05). AD (*N* = 132): Alzheimer's disease; BBS: Berg balance scale; BMI: body mass index; CV: coefficient of variation; DTC: dual‐task cost; DTUG: dual‐task timed up and go test; HC (*N* = 98): healthy controls; SVCI (*N* = 109): subcortical vascular cognitive impairment; TUG: timed up and go test.

When comparing the ad and the SVCI groups, the SVCI group exhibited significantly higher time consumption in the TUG and DTUG, coupled with lower BBS, shorter step length and slower gait speed than the ad group. Additionally, DTC exhibited an increasing trend in the ad group compared to the SVCI group. The CV in gait speed and step length was greater in the SVCI group compared to the ad group (Figure 2).

### 3.3 The Regions of GMV Correlated With the Gait Metrics in ad and SVCI Groups

**FIGURE 3:**
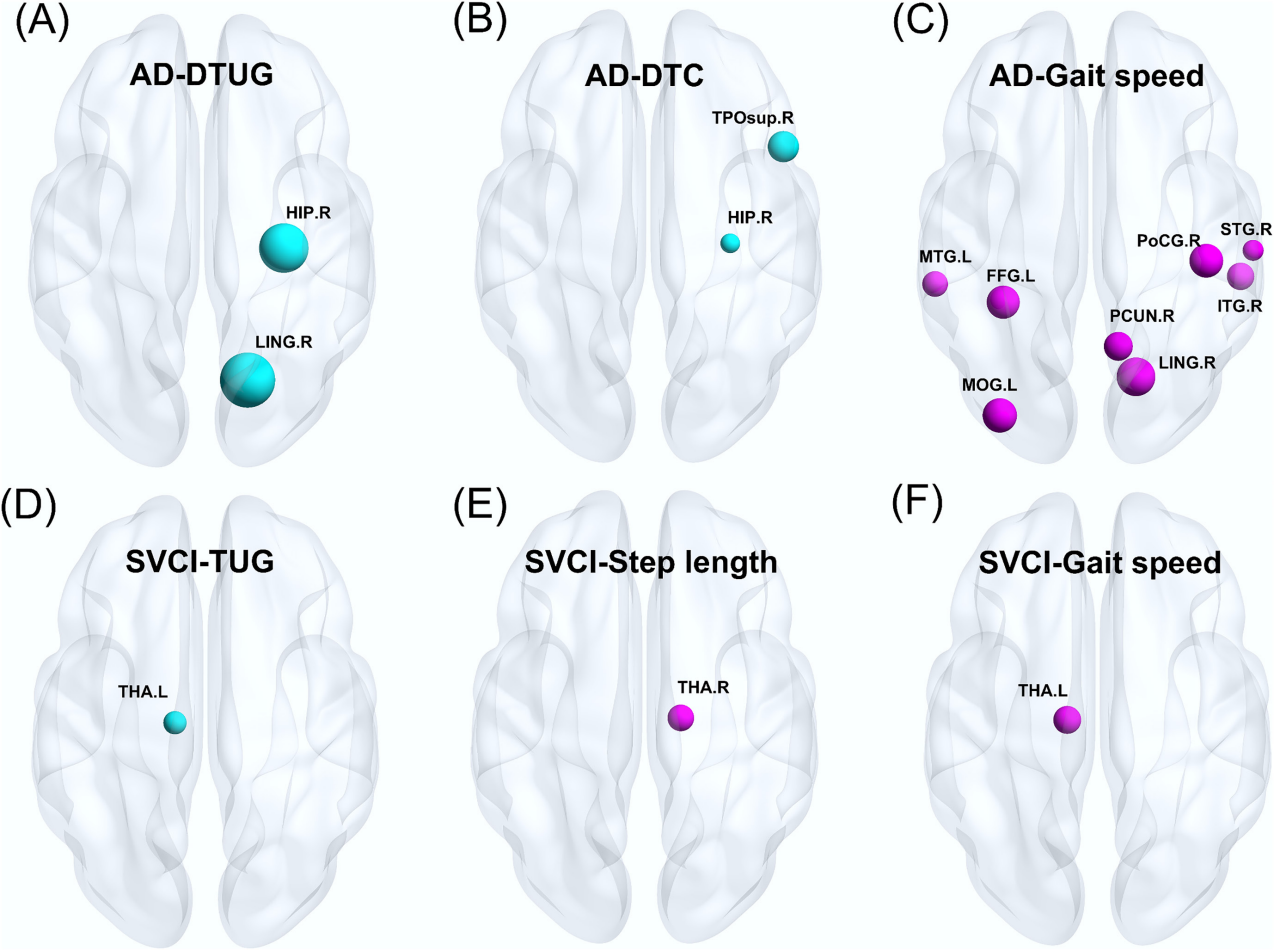
Significant clusters comprising the positively and negatively GMV patterns for gait parameters in (A–C) ad and (D–F) SVCI groups, corrected for age, gender, BMI and TIV (blue ball indicating a negative correlation and red ball indicating a positive correlation, FWE cluster‐level correction, *p <* 0.025). ad: Alzheimer's disease (*N* = 132); BMI: body mass index; DTC: dual‐task cost; DTUG: dual‐task timed up and go test; FFG.L: left fusiform gyrus; GMV: grey matter volume; HIP.R: right hippocampus; ITG.R: right inferior temporal gyrus; LING.R: right lingual gyrus; MOG.L: left middle occipital gyrus; MTG.L: left middle temporal gyrus; PCUN.R: right precuneus; PoCG.R: right postcentral gyrus; STG.R: right superior temporal gyrus; SVCI: subcortical vascular cognitive impairment (*N* = 109); THA.L: left thalamus; THA.R: right thalamus; TIV: total intracranial volume; TPOsup.R: right temporal pole: superior temporal gyrus; TUG: timed up and go test.

In the AD group, gait speed showed positive correlations with GMV in the posterior temporal–parietal–occipital regions, including the left fusiform gyrus, middle occipital lobe and middle temporal gyrus, as well as the right lingual gyrus, postcentral gyrus, precuneus, inferior temporal gyrus and superior temporal gyrus. DTUG demonstrated negative associations with GMV in the right lingual gyrus and hippocampus, while DTC was linked to the GMV in the right hippocampus and temporal pole: superior temporal gyrus (Figure 3A–C and Table S5).

In the SVCI group, TUG exhibited negative correlations with GMV in the left thalamus, whereas gait speed showed a positive correlation with the GMV in the left thalamus. Additionally, step length was positively correlated with GMV in the right thalamus (Figure 3D–F and Table S5).

### 3.4 The Regions of CBF Correlated With the Gait Metrics in ad and SVCI Groups

**FIGURE 4:**
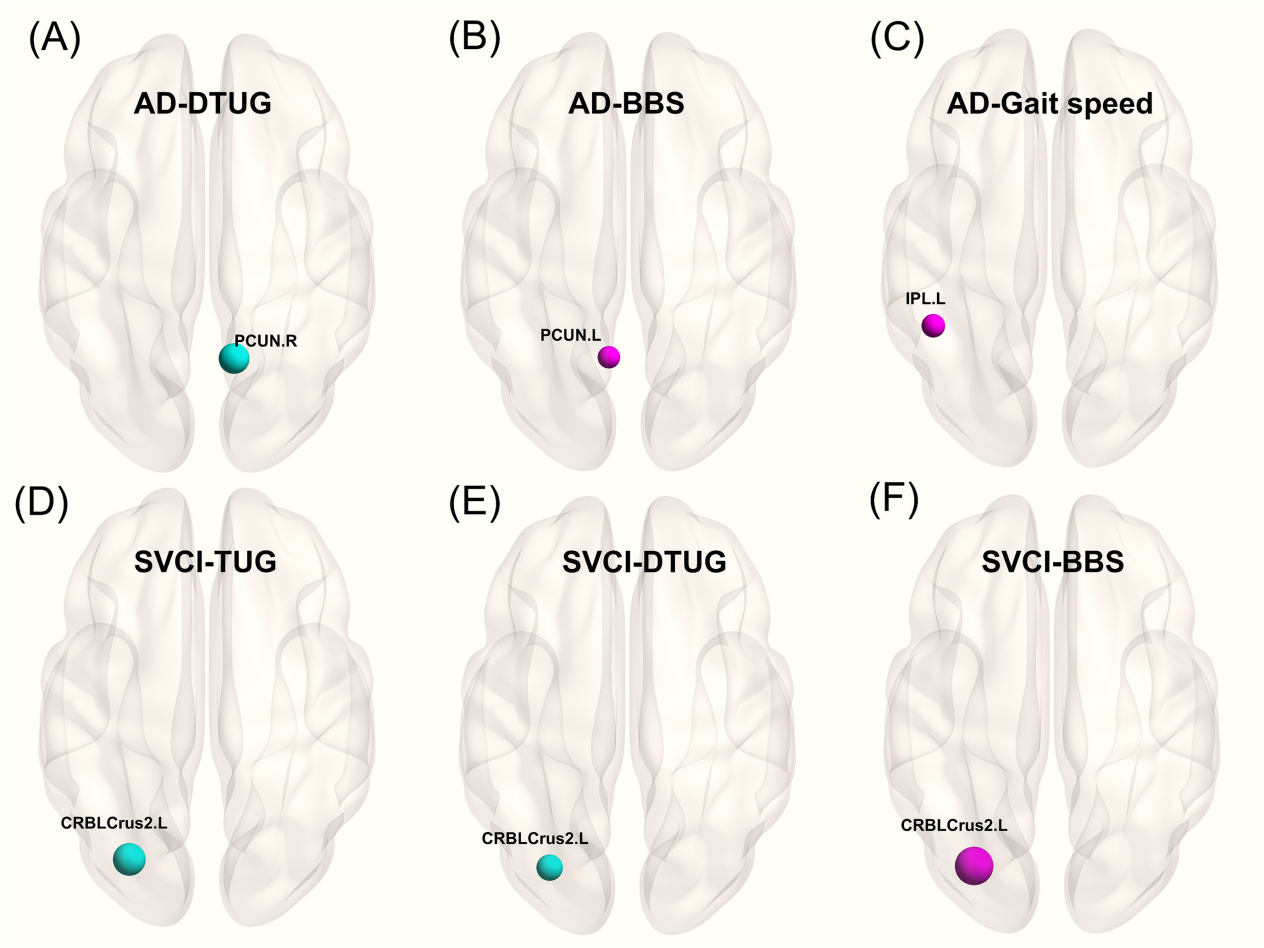
Significant clusters comprising the positively and negatively weighted CBF patterns for gait parameters in (A–C) ad and (D–F) SVCI groups, corrected for age, gender, BMI (blue ball indicating a negative correlation and red ball indicating a positive correlation, FWE cluster‐level correction, *p <* 0.025). ad: Alzheimer's disease (*N* = 132); BBS: Berg balance scale; BMI: body mass index; CBF: cerebral blood flow; CRBLCrus2.L: left cerebellum crus2; CV: the coefficient of variation; DTC: dual‐task cost; DTUG: dual‐task timed up and go test; IPL.L: left inferior parietal gyrus; PCUN.L: left precuneus; PCUN.R: right precuneus; SVCI: subcortical vascular cognitive impairment (*N* = 109); TUG: timed up and go test.

In the ad group, DTUG showed a negative correlation with CBF in the right precuneus, while BBS demonstrated a positive correlation with CBF in the left precuneus. Additionally, gait speed exhibited a positive correlation with CBF in the left inferior parietal lobe (Figure 4A–C and Table S6).

In the SVCI group, both TUG and DTUG displayed negative correlations with CBF in the left crus2 of the cerebellum, whereas BBS was positively correlated with CBF in the same region. (Figure 4D–F and Table S6).

### 3.5 The Regions of GMV and CBF Correlated With the Gait Metrics in ad‐MCI and svMCI Groups

In the ad‐MCI group, DTC was associated with GMV in the right hippocampus and right temporal pole: the superior temporal gyrus (Figure S2A and Table S7). Additionally, CBF in the left posterior cingulate gyrus was linked to DTC (Figure S3A and Table S8). In the svMCI group, gait speed was associated with GMV in the left inferior frontal gyrus (Figure S2B and Table S7). Regarding perfusion, similar to the SVCI group, the CV of gait speed was correlated with CBF in the left crus2 of the cerebellum in the svMCI group (Figure S3B and Table S8). The GMV and CBF regions correlated with gait metrics in the adD and sVD groups are also detailed in Figures S2 and S3 and Tables S7 and S8.

### 3.6 The Comparison of GMV and Perfusion Between MCI Subtypes and HC Groups

**FIGURE 5:**
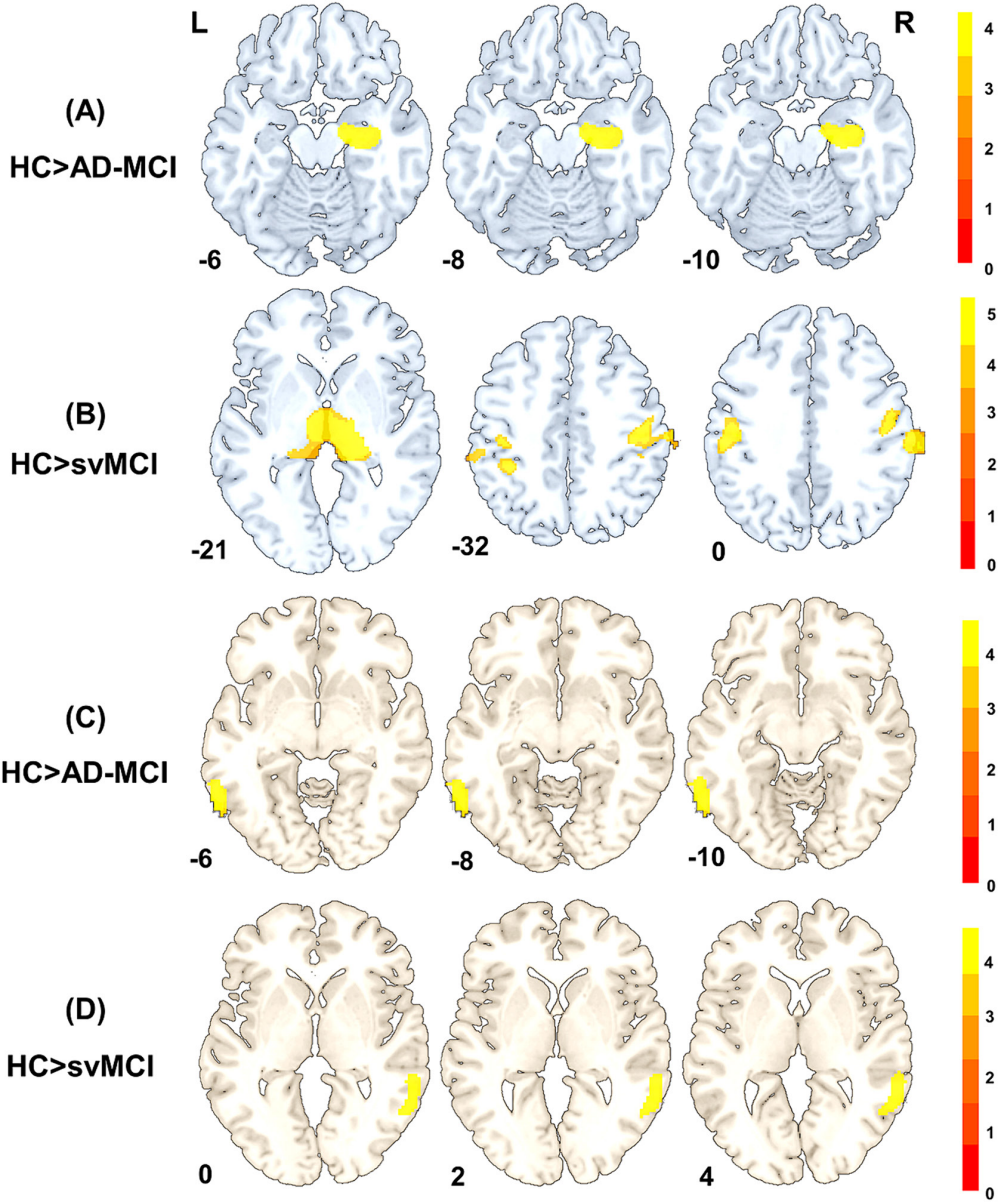
(A) Differences of brain GMV between HC and ad‐MCI, (B) differences of brain GMV between HC and svMCI, (C) differences of brain CBF between HC and ad‐MCI and (D) differences of brain CBF between HC and svMCI (FWE cluster‐level correction, *p <* 0.05). ad‐MCI: mild cognitive impairment due to ad; CBF: cerebral blood flow; GMV: grey matter volume; HC: healthy controls; svMCI: subcortical vascular mild cognitive impairment.

Compared to the HC group, patients with ad‐MCI exhibited significant grey matter atrophy in the right hippocampus (Figure 5A and Table S9), while in patients with svMCI, grey matter atrophy primarily involved the right thalamus and bilateral postcentral gyrus (Figure 5B and Table S9). In terms of perfusion, compared to the HC group, the ad‐MCI group showed reduced CBF in the left inferior temporal gyrus, while the svMCI group showed decreased CBF in the right middle temporal gyrus (Figure 5C,D and Table S9).

### 3.7 The Relationship Analysis and Mediation Analysis in ad‐MCI and svMCI Groups

**FIGURE 6:**
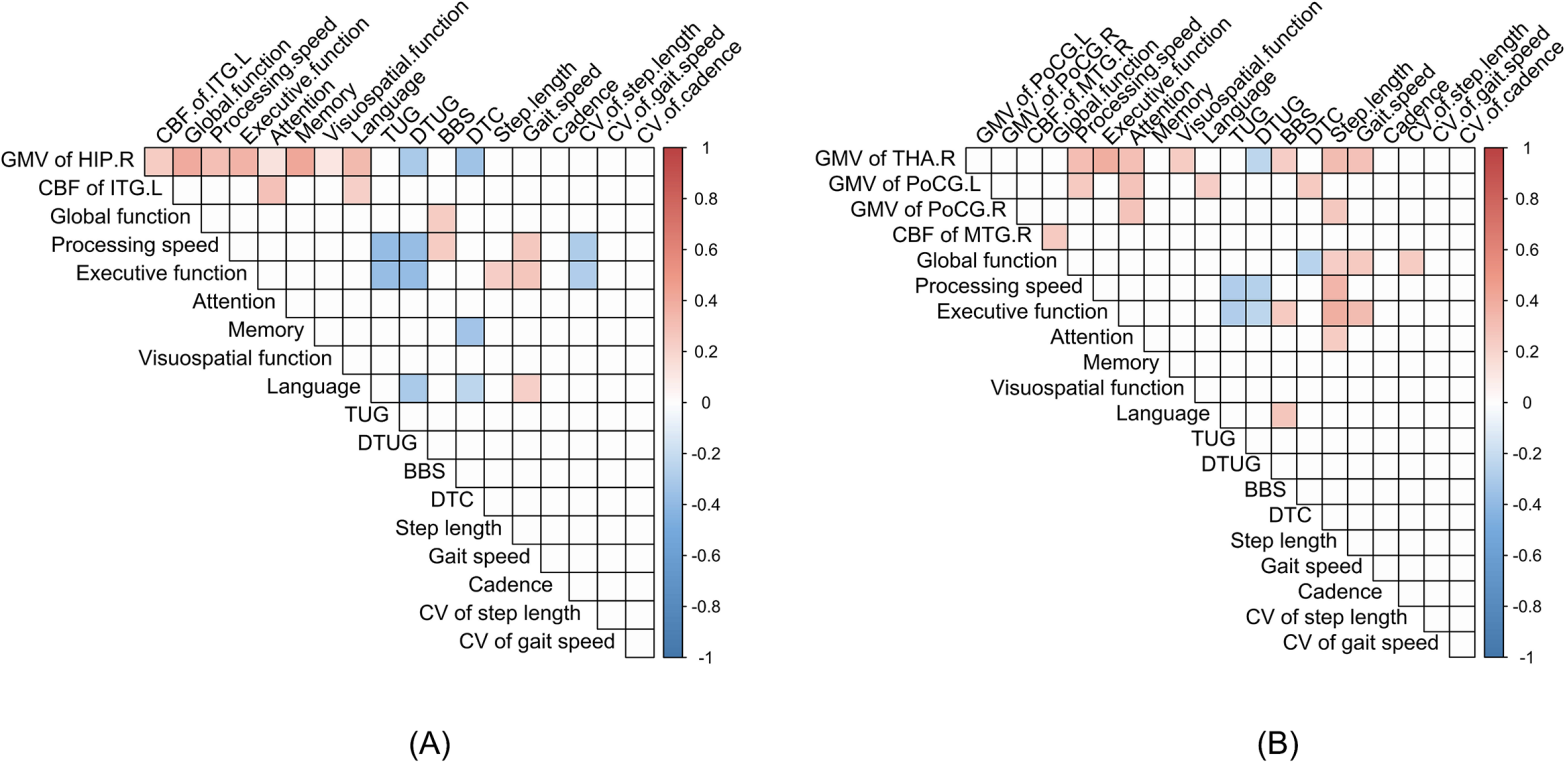
(A) The relationship between brain regions and gait and cognitive function in ad‐MCI group. (B) The relationship between brain regions and gait and cognitive function in svMCI group (blue ball indicating a negative correlation and red ball indicating a positive correlation, FWE cluster‐level correction, *p <* 0.05). ad‐MCI: mild cognitive impairment due to ad; BBS: Berg balance scale; CV: coefficient of variation; DTC: dual‐task cost; DTUG: dual‐task timed up and go test; svMCI: subcortical vascular mild cognitive impairment; TUG: timed up and go test.

In the AD‐MCI group, the GMV of the right hippocampus correlated positively with overall cognitive function, processing speed, executive function and memory. Additionally, the GMV of the right hippocampus was negatively linked to the DTUG and DTC. Furthermore, the DTC demonstrated a negative correlation with memory and language. The TUG and DTUG were negatively associated with processing speed and executive function, while gait speed positively correlated with processing speed and executive function. Regarding gait variability, the CV of step length was positively correlated with processing speed and executive function, and the DTUG was negatively correlated with language (Figure 6A).

In the svMCI group, the GMV of the right thalamus and bilateral postcentral gyrus was linked to attention, with the GMV of the right thalamus positively associated with executive function and processing speed. Gait speed primarily showed a positive correlation with the GMV of the right thalamus, while step length and cadence were positively correlated with the GMV in both the right thalamus and left postcentral gyrus. Additionally, DTC was positively correlated with the GMV in the left postcentral gyrus. TUG and DTC were primarily positively correlated with processing speed and executive function, while gait speed was associated with overall cognitive and executive functions. Conversely, DTC was negatively correlated with global cognitive function (Figure 6B).

**FIGURE 7:**
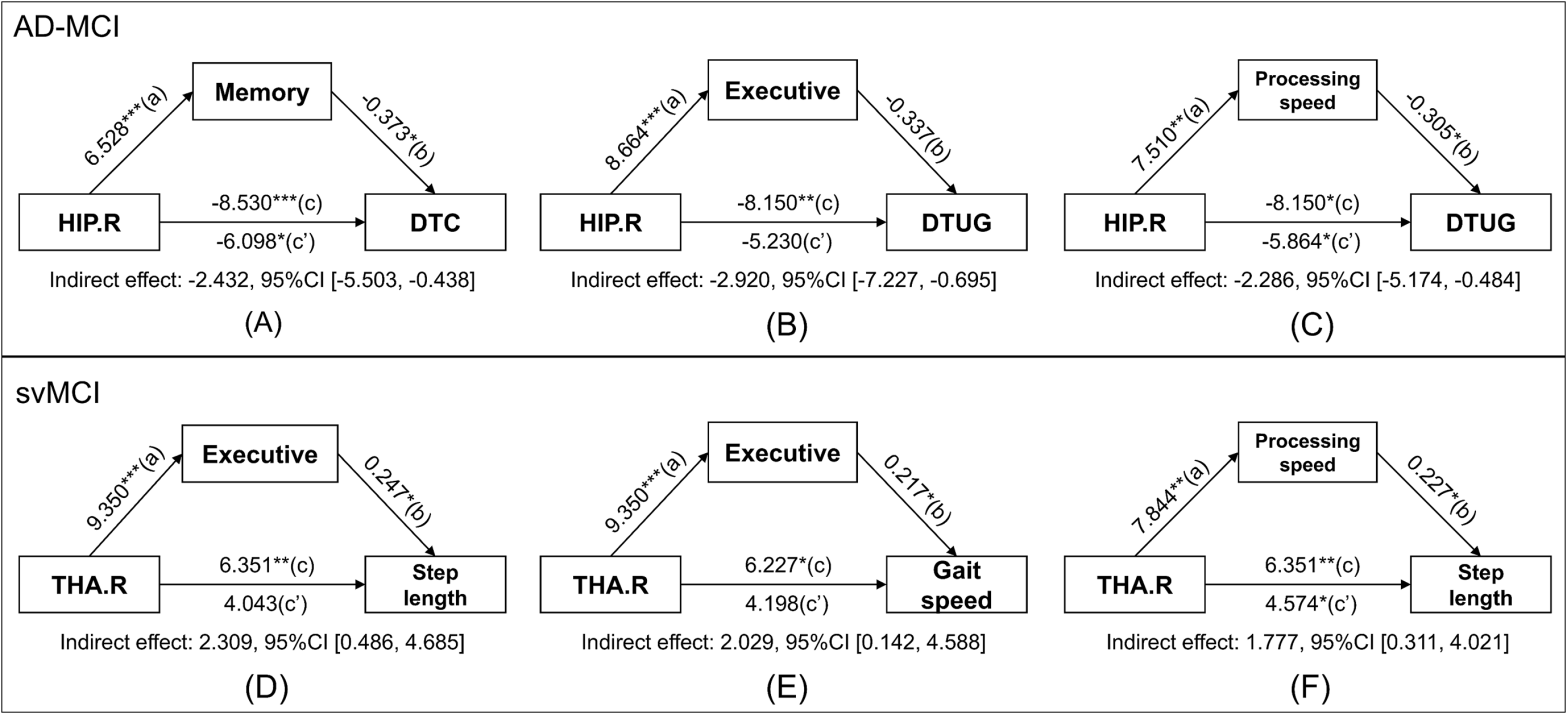
Cognition mediates the relationship of brain regions to gait parameters in (A–C) ad‐MCI group and (D–F) svMCI group, corrected for age, gender, education, BMI and TIV. AD‐MCI: mild cognitive impairment due to AD; BBS: Berg balance scale; BMI: body mass index; CI: confidence interval; DTC: dual‐task cost; DTUG: dual‐task timed up and go test; HIP.R: right hippocampus; svMCI: subcortical vascular mild cognitive impairment; THA.R: right thalamus; TIV: total intracranial volume; TUG: timed up and go test.

Further mediation analysis revealed that memory function mediates the relationship between the GMV of the right hippocampus and DTC (indirect effect: −2.432, 95% CI [−5.503, −0.438]). In contrast, executive function (indirect effect: −2.920, 95% CI [−7.227, −0.695]) and processing speed (indirect effect: −2.286, 95% CI [−5.174, −0.484]) mediate the association between the GMV of the right hippocampus and DTUG in the ad‐MCI group (Figure 7A–C). In the svMCI group, the executive function primarily acts as a mediator in the relationship between the GMV of the right thalamus and gait speed (indirect effect: 2.309, 95% CI [0.486, 4.685]). Executive function (indirect effect: 2.029, 95% CI [0.142, 4.588]) and processing speed (indirect effect: 1.777, 95% CI [0.311, 4.021]) mediate the relationship between the GMV of the right thalamus and step length (Figure 7D–F).

## 4 Discussion

In this study, we first compared gait differences between patients with ad and CSVD, exploring the associated brain structure and CBF alterations. Our primary findings reveal that both ad and CSVD patients exhibit gait disruptions that worsen with cognitive decline. When comparing gait patterns, patients with SVCI showed more pronounced gait disturbances and greater stride variability compared to those with ad. Conversely, ad patients experienced higher costs during dual‐task walking. This distinction is especially evident in the ad‐MCI and svMCI stages but diminishes as patients advance to the dementia stage.

Walking in daily environments is a complex task that involves higher order neural processes and cognition. Cognitive impairment is a key feature of ad and CSVD. ad primarily presents with memory deficits, while CSVD is marked by deficiencies in executive functions and processing speed [16]. Despite these differences, both conditions are associated with gait disturbances, aligning with previous studies that link gait disruptions to cognitive decline [17, 18]. Notably, across all subgroups, patients with SVCI consistently show the slowest gait speed, shortest step length and slowest cadence, even when compared to patients with ad. This finding supports prior research indicating that individuals with non‐MCI/non‐ad dementia tend to have more noticeable gait disturbances than those with amnestic MCI/ad [2, 5]. As cognitive decline progresses owing to neuropathological or vascular lesions, these pathological impairments likely lead to widespread effects on brain regions involved in walking, including those responsible for sensory integration, motor planning and movement coordination [19]. In addition, the SVCI group exhibited more diverse walking patterns and higher gait variability, beyond common gait abnormalities, indicating inconsistencies in walking rhythms and challenges in maintaining steady gait due to compromised motor control [20]. Gait variability, which reflects fluctuations in walking rhythm and balance control, is notably influenced by sustained attention [21]. Attention plays a crucial role in gait control, with simultaneous cognitive tasks potentially disrupting gait patterns [22]. Increased gait variability may predict changes in sustained attention among CSVD patients, highlighting attention's critical role in locomotion. Although fall rates between ad and SVCI have not been directly compared, research on hip fracture surgery revealed a 36% fall rate for ad and 59% for VD among patients with dementia, possibly owing to the more severe brain damage in VD [23]. Across all subgroups, patients with ad demonstrated the highest DTC compared to the other groups. Specifically, the ad‐MCI group showed a similar increase in DTC compared to the svMCI group during the DTUG test. Given these findings, it is reasonable to suspect that patients with ad experience greater cognitive demands compared to those with vascular cognitive impairment, possibly indicating a decline in the coordination between cognitive and motor functions.

To understand the mechanisms behind gait differences between ad and CSVD, we further examined brain structure and perfusion. In AD, gait speed primarily correlated with GMV in the posterior temporal–parietal–occipital brain regions, including the lingual gyrus, fusiform gyrus, middle occipital lobe, postcentral gyrus, precuneus, inferior temporal gyrus and superior temporal gyrus. These regions are primarily responsible for visual perception, language, semantic memory processing and multimodal sensory integration [24, 25, 26]. Studies on CBF patterns have indicated a decrease from the precuneus, posterior cingulate and temporal–parietal regions to broader areas from HC to MCI to ad [ 27]. These brain regions are key in coordinating motor behaviour and attention during walking, which might be overactive in older adults with gait issues [28]. Connectivity disruptions in the occipital lobe areas linking motor, visual and cognitive processing could affect visuospatial abilities during walking [29], while the precuneus, a part of the default mode network (DMN), crucial for attention shifts, guides focus towards relevant information [28]. Hence, atrophy in the posterior brain regions may contribute to the reduced gait speed observed in patients with ad. The DTUG exhibited associations with the GMV in the temporal‐occipital lobe, including the hippocampus and lingual gyrus, aligning with a previous study suggesting a pattern specific to dual‐task gait [30]. The hippocampus, integral to spatial navigation and a component of the corticocerebellar network, is essential for developing cognitive strategies and motor routines that underlie the execution of various movements [31]. Atrophy of the hippocampus may account for uncoordinated gait and locomotor functional decline. The DTC showed a close relationship with the GMV in right temporal pole: superior temporal gyrus and hippocampus of the ad group. The DTC has been suggested to correlate with cognitive impairment, even in individuals with a relatively healthy gait [9]. Severer cognitive impairment may partly explain the more pronounced increased DTC in the ad group than in the SVCI group. Previous studies have also shown an association of superior temporal gyrus with dynamic gait tasks and spatial gait variability in older individuals, attributed to the intact vestibular function of this region [32]. In line with these observations, the elevated gait variability, coupled with reduced gait speed, may lead to greater energy expenditure, balance difficulties and an increased risk of falls [33, 34]. These factors may be linked to reduced hippocampal volume and cognitive impairment [35], providing additional support for our findings regarding the association between hippocampal atrophy and impaired gait dynamics.

Regarding perfusion, significant correlations between DTUG, BBS and CBF in the precuneus suggested lower perfusion in the precuneus, longer DTUG times and poorer BBS performance. Furthermore, gait speed correlated with the CBF in the inferior parietal lobe in ad group. The inferior parietal lobe is vital in integrating perceptual spatiotemporal information, and functional impairments in this area could result in disrupted control and bilateral gait incoordination [36]. This could explain why patients with ad experience momentary inability to move despite intending to walk, resembling symptoms seen in PD [37].

In patients with SVCI, the TUG test, gait speed and step length were predominantly linked to the GMV in the thalamus, consistent with prior studies indicating that lesions in the thalamus contribute to slower gait speed and increased variability in patients with ad experiencing WMHs [21]. The thalamus is a pivotal subcortical brain region in motor movement networks, contributing to gait and posture control through connections to the brain stem. It also collaborates with the frontal cortex in intricate motor and cognitive functions [38]. In terms of perfusion, both TUG and DTUG were negatively correlated with CBF in the left crus2 of the cerebellum, and BBS was positively correlated with CBF in the same region. The cerebellum, crucial for motor control and cognitive functions, interfaces with diverse brain networks, influencing language, memory and visuospatial skills [39]. Unlike in ad, gait‐related CBF variation was primarily concentrated in the cerebellum, indicating that reduced cerebellar perfusion may contribute to gait disorders and balance issues. This may be due to the extensive connections between the cerebellum and cortical regions, such as the frontal lobe, involved in gait coordination [21].

To investigate gait patterns alongside brain structure and perfusion alterations in the early MCI stage of ad and CSVD, we compared patients with ad‐MCI and svMCI. The differences between these subtypes were more pronounced than those between adD and sVD subtypes. In the ad‐MCI group, DTC was associated with GMV in the temporal lobe and with CBF in the left posterior cingulate gyrus, a key node of DMN. Considering GMV alterations in ad, it indicated that atrophy in the temporal lobe contributed to early gait disorders in pre‐ad stages, extending to parietal and occipital lobes in ad stages. Alterations in CBF within DMN have been observed during the ad period and can also be detected in the MCI stage, suggesting that the perfusion alteration in DMN might serve as a sensitive biomarker for gait disturbances in the early stages of cognitive impairment. Similar to SVCI, increased gait variability in speed was associated with reduced cerebellar CBF in the svMCI phase, suggesting that decreased CBF in the left cerebellum contributes to increased gait variability in the early MCI phase, ultimately leading to poorer performance in standard gait tests as the condition worsens.

Comparing brain structure and perfusion in the different MCI subtypes and HC groups revealed some specific patterns: ad‐MCI exhibited right hippocampal atrophy, while svMCI exhibited thalamic and postcentral gyrus atrophy. Both subtypes of MCI showed reduced CBF in the temporal lobe compared to HC. In the ad‐MCI group, hippocampal volume was correlated with multidomain cognitive function. In contrast, in the svMCI group, thalamic volume was correlated with attention, executive function, processing speed and gait metrics. Executive function and processing speed mediate the linking of brain structure alterations to gait metrics in svMCI. However, in ad‐MCI, in addition to executive function and processing speed, memory function is a mediator between hippocampal volume and the cost of performing a dual gait task. These findings highlight shared neural connections between cognitive decline and gait disorders, shedding light on similarities and distinct cognitive control mechanisms influencing gait in ad‐MCI and svMCI.

This study provides the first comparative analysis of gait disorders in patients with ad and CSVD, revealing alterations in brain structure and cerebral perfusion influencing these gait disorders. However, this study has some limitations. First, our ad diagnosis relied on the 2011 NIA‐AA criteria rather than the 2018 ‘ATN’ diagnostic criteria because most participants lacked cerebrospinal fluid and positron emission tomography imaging. Second, despite a large sample size for imaging studies, our cross‐sectional design precludes causal conclusions. Future longitudinal studies would be advantageous in better understanding the evolving patterns and causal connections between alterations in brain structure and perfusion alongside cognitive and gait alterations. Finally, as gait and cognitive control depend on intricate neural networks, relying solely on structural and perfusion MRI might limit detecting subtle associations, especially after accounting for multiple covariates. Further functional research may better elucidate the distinct gait characteristics of ad and CSVD by examining synchronous alterations in distant brain regions.

In conclusion, patients with CSVD experiencing cognitive impairment exhibit more pronounced gait impairments and stride variability, while patients with ad show higher costs in dual‐task walking. Gait speed primarily correlates with volume in posterior temporal–parietal–occipital areas in patients with ad, contrasting with thalamic volume associations in SVCI. CBF alterations are consistently located in posterior brain regions in ad, although to a lesser extent than volumetric changes. In patients with SVCI, CBF in the cerebellum correlates with TUG performance and balance. Similar patterns are observed in the MCI stages, where cognitive impairment mediates the relationship between hippocampal and thalamic volumes and gait disturbances in ad‐MCI and svMCI, respectively. This study unveils the distinct characteristics of gait disorders and the underlying mechanisms in brain structure and perfusion among patients with AD and CSVD. These findings hold significance for the early detection of gait disorders in ad and CSVD by identifying specific regions undergoing structural and perfusion alterations.

## Ethics Statement

All participants provided written informed consent, and the research ethics committee of the First Affiliated Hospital of Anhui Medical University approved this study (reference number: Quick‐PJ 2023‐14‐76).

## Conflicts of Interest

The authors declare no conflicts of interest.

## Supporting information


**Table S1** Common English abbreviations.
**Table S2** Abbreviations of all brain regions in figures.
**Table S3** Summary of multicomparison correction methods.
**Table S4** Presentation of raw gait parameters among different subgroups.
**Table S5** Coordinates of brain regions in the positively and negatively GMV patterns for gait parameters in ad and SVCI groups.
**Table S6** Coordinates of brain regions in the positively and negatively CBF patterns for gait parameters in ad and SVCI groups.
**Table S7** Coordinates of brain regions in the GMV for gait parameters in the ad‐MCI, svMCI, adD and sVD groups.
**Table S8** Coordinates of brain regions in the CBF patterns for gait parameters in the ad‐MCI, svMCI, ADD and sVD group.
**Table S9** Coordinates of brain regions in the difference of GMV and CBF in AD‐MCI, svMCI and HC groups.
**Figure S1** Flow chart of inclusion and exclusion criteria.
**Figure S2** Significant clusters comprising the GMV patterns for gait parameters in the ad‐MCI, svMCI, adD and sVD groups.
**Figure S3** Significant clusters comprising the CBF patterns for gait parameters in the ad‐MCI, svMCI, ADD and sVD groups.
