# Supplementary material for: Distinctive Gait Variations and Neuroimaging Correlates in Alzheimer's Disease and Cerebral Small Vessel Disease
Source: J Cachexia Sarcopenia Muscle. 2024 Nov 17;15(6):2717–28. doi: 10.1002/jcsm.13616 (PMC11634515; doi:10.1002/jcsm.13616)
Supplement: Supplementary file 1 — Table S1 Common English abbreviations. Table S2 Abbreviations of all brain regions in figures. Table S3 Summary of multicomparison correction methods. Table S4 Presentation of raw gait parameters among different subgroups. Table S5 Coordinates of brain regions in the positively and negatively GMV patterns for gait parameters in ad and SVCI groups. Table S6 Coordinates of brain regions in the positively and negatively CBF patterns for gait parameters in ad and SVCI groups. Table S7 Coordinates of brain regions in the GMV for gait parameters in the ad‐MCI, svMCI, adD and sVD groups. Table S8 Coordinates of brain regions in the CBF patterns for gait parameters in the ad‐MCI, svMCI, ADD and sVD group. Table S9 Coordinates of brain regions in the difference of GMV and CBF in AD‐MCI, svMCI and HC groups. Figure S1 Flow chart of inclusion and exclusion criteria. Figure S2 Significant clusters comprising the GMV patterns for gait parameters in the ad‐MCI, svMCI, adD and sVD groups. Figure S3 Significant clusters comprising the CBF patterns for gait parameters in the ad‐MCI, svMCI, ADD and sVD groups. [file JCSM-15-2717-s001.docx]

**Supplemental Materials for**

**Distinctive Gait Variations and Neuroimaging Correlates in Alzheimer’s Disease and Cerebral Small Vessel Disease**

**Authors**

Xia Zhou^1#^, Wen-Wen Yin^2#^, Chao-Juan Huang^1#^, Si-Lu Sun^1^, Zhi-Wei Li^1^, Ming-Xu Li^1^, Meng-Meng Ren^1^, Ya-Ting Tang^1^, Jia-Bin Yin^1^, Wen-Hui Zheng^1^, Chao Zhang^3^, Yu Song^4^, Ke Wan^1^, Yue Sun^5^, Xiao-Qun Zhu^1*^, Zhong-Wu Sun^1*^

**Affiliations**

^1^ Department of Neurology, the First Affiliated Hospital of Anhui Medical University, Hefei, China

^2^ Department of Rehabilitation Medicine, the First Affiliated Hospital of Anhui Medical University, Hefei, China

^3^ Department of Neurology, the First Affiliated Hospital of USTC, Hefei, China

^4^ Department of Radiology, the First Affiliated Hospital of Anhui Medical University, Hefei, China

^5^ Department of Radiology, the Second Affiliated Hospital of Anhui Medical University, Hefei, China

^#^ These authors contributed equally to this work.

*** Corresponding Author**

Xiao-Qun Zhu MD., Ph.D. Professor of Neurology

Department of Neurology, the First Affiliated Hospital of Anhui Medical University, 218 Jixi Road, Hefei, Anhui Province 230022

Tel: 86-10-62922328

Email: [zxq_ayfy@163.com](mailto:zxq_ayfy@163.com)

Zhongwu Sun MD., Ph.D. Professor of Neurology

Department of Neurology, the First Affiliated Hospital of Anhui Medical University, 218 Jixi Road, Hefei, Anhui Province 230022

Tel: 86-10-62922328

Email: sunzhwu@126.com

Content

[Method 3](#_Toc175341545)

[Inclusion criteria for AD, CSVD and HC 3](#_Toc175341546)

[Exclusion criteria for AD and CSVD 3](#_Toc175341547)

[MRI acquisition 4](#_Toc175341548)

[Voxel-Based Morphometry (VBM) analysis and CBF Analysis 4](#_Toc175341549)

[Abbreviation 6](#_Toc175341550)

[Table *S1* Common English abbreviations. 6](#_Toc175341551)

[Table *S2* Abbreviations of all brain regions in figures 7](#_Toc175341552)

[Results 8](#_Toc175341553)

[Table *S3* Summary of multi-comparison correction methods 8](#_Toc175341554)

[Table *S4* Presentation of raw gait parameters among different subgroups 9](#_Toc175341555)

[Table *S5* Coordinates of brain regions in the positively and negatively GMV patterns for gait parameters in AD and SVCI groups 10](#_Toc175341556)

[Table *S6* Coordinates of brain regions in the positively and negatively CBF patterns for gait parameters in AD and SVCI groups 11](#_Toc175341557)

[Table *S7* Coordinates of brain regions in the GMV for gait parameters in the AD-MCI, svMCI, ADD and sVD groups 12](#_Toc175341558)

[Table *S8* Coordinates of brain regions in the CBF patterns for gait parameters in the AD-MCI, svMCI, ADD and sVD group 13](#_Toc175341559)

[Table *S9* Coordinates of brain regions in the difference of GMV and CBF in AD-MCI, svMCI and HC groups 14](#_Toc175341560)

[Fig. *S1* Flow chart of inclusion and exclusion criteria 15](#_Toc175341561)

[Fig. *S2* Significant clusters comprising the GMV patterns for gait parameters in the AD-MCI, svMCI, ADD and sVD groups 16](#_Toc175341562)

[Fig. *S3* Significant clusters comprising the CBF patterns for gait parameters in the AD-MCI, svMCI, ADD and sVD groups 17](#_Toc175341563)

[References 18](#_Toc175341564)

**Method**

**Inclusion criteria for** **AD, CSVD and HC**

The mild cognitive impairment (MCI) due to probable Alzheimer’s disease (AD-MCI) group’s inclusion criteria comprised: 1) self-reported cognitive complaints by participants or informants; 2) Montreal cognitive assessment (MoCA) scores^1^: illiterate individuals ≤13, those with 1–6 years of education ≤ 19, and those with 7 or more years of education≤ 24; 3) clinical dementia rating (CDR) = 0.5; 4) Hachinski ischemic scale score < 4 points; 5) preserved capacity for daily living. Probable Alzheimer’s disease dementia (ADD) diagnosis followed the diagnostic and statistical manual of mental disorders, fourth edition (DSM-IV) criteria^2^, with CDR = 1 or 2.

Cerebral small vessel disease (CSVD) inclusion criteria were: 1) presence of at least one atherosclerosis risk factor (e.g., smoking, drinking, hypertension, diabetes mellitus, hyperlipidemia, or cardiac disease); 2) MRI findings consistent with CSVD imaging characteristics, including white matter hyperintensities^3^ (WMHs) (periventricular WMHs scores ≥ 3 or deep WMHs scores 2–3 according to Fazekas rating scales), lacunes ≥ 1, perivascular space (PVS) ≥ grade 2^4^, and cerebral microbleeds (CMBs) ≥ 1. Similarly, CSVD was further classified into subtypes—subcortical non-cognitive impairment (sNCI), subcortical vascular mild cognitive impairment (svMCI)，and subcortical vascular dementia (sVD). svMCI was diagnosed according to the following criteria: 1) MoCA scores: illiterate individuals ≤13, those with 1–6 years of education ≤ 19, and those with 7 or more years of education≤ 24; 2) CDR = 0.5, sVD met the following criteria: 1) following DSM-IV criteria, 2) CDR = 1 or 2. The CSVD patients experiencing cognitive impairment were termed as the subcortical vascular cognitive impairment (SVCI) group.

HC met the following criteria: 1) no reported memory or other cognitive complaints; 2) MoCA scores within the normal range, adjusted for educational levels; 3) normal brain MRI based on traditional non-CSVD criteria; 4) absence of significant heart, liver, kidney, lung, or other systemic diseases; and 5) no history of tumor-related illnesses.

**Exclusion criteria for AD and CSVD**

Exclusion criteria for AD and CSVD included: 1) history of stroke, tumors, brain trauma, or intracranial surgery; 2) cognitive impairment secondary to other neuropsychiatric disorders (e.g., frontotemporal dementia, dementia with Lewy bodies, Parkinson’s disease, severe depression, schizophrenia, encephalitis, epilepsy and other neurological/psychiatric illness); 3) systemic diseases (e.g., thyroid dysfunction, syphilis, and HIV); 4) CSVD secondary to infection, autoimmune inflammation, tumor, trauma, poisoning, radiation, metabolic encephalopathy, heredity, or cerebral amyloid angiopathy; 5) severe dementia preventing cooperation with MRI; 6) large acute infarction area (diameter > 2 cm); 7) WMHs (Fazekas grade ≥ 2), lacunes, CMBs or PVS in the AD-MCI/ADD group; 8) inadequate MRI quality; 9) severe visuospatial deficits, hearing impairments, or language disorders; in vivo dentures or metallic stents; and 10) serious orthopedic and muscle diseases or a history of surgery involving the lower limbs.

**MRI acquisition**

MRI data were collected using a 3.0-Tesla MRI scanner (Discovery MR750w, GE, Milwaukee, WI) equipped with a 24-channel head coil. Three-dimensional (3D) T1-weighted images were acquired with the following parameters: repetition time (TR) = 8.5 ms, echo time (TE) = 3.2 ms, flip angle (FA) = 12°, field of view (FOV) = 256 × 256 mm², matrix size = 256 × 256, slice thickness = 1.0 mm, and acquisition time = 296 s. Pseudocontinuous arterial spin labeling (PCASL) parameters included TR = 5,070 ms, TE = 11.48 ms, FA = 111°, FOV = 240 mm × 240 mm², matrix size = 128 × 128, slice thickness = 3.0 mm, post labeling delay time = 2,025 ms, 50 slices covering the entire brain, and acquisition time = 294 s. Additional brain sequences comprised T2-FLAIR with parameters TR = 9,000 ms, TE = 119.84 ms, FA = 160°, FOV = 225 × 225 mm², matrix size = 512 × 512, 19 contiguous 7-mm-thick axial slices, and acquisition time = 117 s. Susceptibility-weighted imaging (SWI) parameters were TR = 45.4 ms, TE = 23.536 ms, FA = 20°, FOV = 240 × 240 mm², matrix size = 512 × 512, slice thickness = 1.0 mm, 138 contiguous slices, and acquisition time = 231 s.

**Voxel-Based Morphometry (VBM) analysis and Cerebral Blood Flow (CBF) Analysis**

VBM analysis was performed with the Computational Anatomy Toolbox 12 (CAT12) (http://www.neuro.uni-jena.de/cat/). The 3D-T1 images were initially segmented into grey matter volume (GMV), white matter volume (WMV), and cerebrospinal fluid (CSF). Subsequently, these segments underwent iterative registration via the Diffeomorphic Anatomical Registration Through Exponentiated Lie Algebra (DARTEL) toolbox. The GMV images were then normalized and modulated into the standard Montreal Neurological Institute (MNI) space. Finally, the modulated spatial normalized GMV images were smoothed with an 8-mm full-width at half maximum kernel (FWHM). Quality checks were performed on the smoothed GMV images using the CAT12 toolbox. The total intracranial volume (TIV) was calculated as the sum of GMV, WMV, and CSF volumes.

CBF maps were automatically generated by software (AW Server, GE Healthcare). PCASL difference image was computed after subtracting the label picture from the control image. Proton density-weighted reference pictures and 3D PCASL difference images were then used to calculate CBF maps, following methods detailed in a previous paper^5^. The downloaded CBF data was analyzed using SPM8 (https://www. fil.ion.ucl.ac.uk/spm/software/spm8/). The CBF map underwent a two-step normalization using the MNI PET template picture. To enhance uniformity, a mean division approach was applied, where the CBF of each voxel was divided by the whole brain mean. After standardization, an 8 mm FWHM smoothed the CBF map.

During the process of VBM and CBF analysis, Family-wise error (FWE) correction at the cluster level addressed multiple comparisons, setting the criterion for statistical significance at *P* < 0.05, adjusted to *P* = 0.001 at the voxel level^6^. Data processing and analysis for brain imaging (DPABI) software (https://rfmri.org/dpabi) was used to extract GMV values from specific structurally changed brain regions and CBF values from specific brain regions for region-of-interest analysis^7^.

**Abbreviation**

**Table *S1* Common English abbreviations.**

| **Full name** | **Abbreviation** |
| --- | --- |
| AD dementia | ADD |
| Alzheimer’s disease | AD |
| Amnestic mild cognitive impairment | aMCI |
| Auditory verbal learning test | AVLT |
| Berg balance scale | BBS |
| Body mass index | BMI |
| Cerebral blood flow | CBF |
| Cerebral small vessel disease | CSVD |
| Clinical dementia rating | CDR |
| Clock drawing task | CDT |
| Coronary artery disease | CAD |
| Coefficient of variation | CV |
| Default mode network | DMN |
| Digit span test | DST |
| Dual-task cost | DTC |
| Dual-task timed up and go test | DTUG |
| Family-wise error | FWE |
| Healthy controls | HC |
| Intelligent device for energy expenditure and activity | IDEEA |
| Magnetic resonance imaging | MRI |
| MCI due to probable AD | AD-MCI |
| Mini-mental state examination | MMSE |
| Montreal cognitive assessment | MoCA |
| Parkinson’s disease | PD |
| Stroop color-word test-A | SCWT-A |
| Stroop color-word test-B | SCWT-B |
| Stroop color-word test-C | SCWT-C |
| Subcortical vascular non-cognitive impairment | sNCI |
| Subcortical cognitive impairment | SVCI |
| Subcortical vascular mild cognitive impairment | svMCI |
| Subcortical vascular dementia | sVD |
| Timed up and go test | TUG |
| Trail making test-A | TMT-A |
| Trail making test-B | TMT-B |
| Total intracranial volume | TIV |
| Vascular dementia | VD |
| White matter hyperintensities | WMHs |

**Table *S2* Abbreviations of all brain regions in figures**

| **Full name** | **Abbreviation** |
| --- | --- |
| Left cerebellum 4_5 | CRBL45.L |
| Left cerebellum crus2 | CRBLCrus2.L |
| Left cerebellum8 | CRBL8.L |
| Left cerebellum9 | CRBL9.L |
| Left fusiform gyrus | FFG.L |
| Left inferior frontal gyrus, triangular part | IFGtri.L |
| Left inferior parietal gyrus | IPL.L |
| Left inferior temporal gyrus | ITG.L |
| Left lingual gyrus | LING.L |
| Left middle occipital gyrus | MOG.L |
| Left postcentral gyrus | PoCG.L |
| Left posterior cingulate gyrus | PCG.L |
| Left precuneus | PCUN.L |
| Left supramarginal gyrus | SMG.L |
| Left superior parietal gyrus | SPG.L |
| Left superior temporal gyrus | STG.L |
| Left thalamus | THA.L |
| Right cerebellum crus2 | CRBLCrus2.R |
| Right hippocampus | HIP.R: |
| Right hippocampus | HIP.R |
| Right inferior temporal gyrus | ITG.R |
| Right lingual gyrus | LING.R |
| Right middle temporal gyrus | MTG.R |
| Right postcentral gyrus | PoCG.R |
| Right precuneus | PCUN.R |
| Right superior parietal gyrus | SPG.R |
| Right temporal pole: superior temporal gyrus | TPOsup.R |
| Right thalamus | THA.R |

**Results**

**Table *S3* Summary of multi-comparison correction methods**

| Table 1 | Bonferroni correction |
| --- | --- |
| Fig. 1 | Bonferroni correction |
| Fig. 2 | Bonferroni correction |
| Fig. 3 | FWE cluster-level correction, *P*<0.05/2 |
| Fig. 4 | FWE cluster-level correction, *P*<0.05/2 |
| Fig. 5 | FWE cluster-level correction, *P*<0.05 |
| Fig. 6 | None multiple comparisons |
| Fig. 7 | Multiple comparison corrections not involved |
| Fig. *S1* | Multiple comparison corrections not involved |
| Fig. *S2* | FWE cluster-level correction, *P*<0.05/4 |
| Fig. *S3* | FWE cluster-level correction, *P*<0.05/4 |

**Table *S4* Presentation of raw gait parameters among different subgroups**

|  | HC (N=82) | AD-MCI (N=98) | ADD(N=34) | sNCI(N=76) | svMCI(N=78) | sVD(N=31) | *P* values |
| --- | --- | --- | --- | --- | --- | --- | --- |
| TUG | 9.04±1.35 | 10.02±2.05 | 12.39±2.77 | 10.28±2.8 | 12.54±5 | 13.93±3.22 | <0.001 |
| DTUG | 9.92±1.58 | 11.98±2.82 | 14.95±3.2 | 11.87±3.32 | 14.88±6.28 | 16.66±3.83 | <0.001 |
| BBS | 55.93±0.38 | 55.04±2.23 | 52.68±4.85 | 54.71±2.75 | 52.17±5.97 | 52.03±5.78 | <0.001 |
| DTC | 9.8±8.32 | 19.49±12.76 | 22.21±15.74 | 15.9±14.21 | 18.75±12.9 | 20.74±18.14 | <0.001 |
| Step length | 0.53±0.07 | 0.5±0.07 | 0.46±0.08 | 0.51±0.08 | 0.44±0.08 | 0.45±0.08 | <0.001 |
| Gait speed | 1.02±0.15 | 0.92±0.17 | 0.81±0.17 | 0.94±0.19 | 0.8±0.18 | 0.78±0.19 | <0.001 |
| Cadence | 109.57±10.65 | 106.97±9.45 | 103.28±10.63 | 106.11±10.4 | 104.6±11.51 | 105.18±12.46 | 0.085 |
| CV of step length | 16.8±6.31 | 14.62±6.14 | 15.29±5.09 | 17.77±8.91 | 17.33±7.5 | 19.09±7.69 | 0.018 |
| CV of gait speed | 22.54±11.53 | 21.36±10.2 | 22.42±10.5 | 24.84±13.7 | 26.7±17.83 | 30.58±17.56 | 0.049 |
| CV of cadence | 12.47±7.86 | 12.3±8.9 | 12.09±5.79 | 14.01±9.75 | 14.37±12.32 | 16.83±13.3 | 0.305 |

HC: health controls, AD-MCI: mild cognitive impairment due to probable Alzheimer’s disease, ADD: probable Alzheimer’s disease dementia, sNCI: subcortical vascular non-cognitive impairment, svMCI: subcortical vascular mild cognitive impairment, sVD: subcortical vascular dementia, TUG: timed up and go test, DTUG: dual-task timed up and go test, BBS: berg balance scale, DTC: dual-task cost, CV: coefficient of variation.

Table *S5* Coordinates of brain regions in the positively and negatively GMV patterns for gait parameters in AD and SVCI groups

| Group-gait metrics |  | Brain regions | Cluster size  (voxel) | MNI coordinate | | | *F* values |
| --- | --- | --- | --- | --- | --- | --- | --- |
|  |  |  |  | X | Y | Z |  |
| AD-DTUG | Cluster1 | LING.R | 1712 | 23 | -60 | -2 | 9.86 |
|  | Cluster2 | HIP.R | 1909 | 38 | -23 | -17 | 8.84 |
| AD-DTC | Cluster1 | TPOsup.R | 1360 | 54 | 18 | -15 | 5.58 |
|  | Cluster2 | HIP.R | 231 | 29 | -6 | -20 | 3.51 |
| AD-Gait speed | Cluster1 | LING.R | 539 | 24 | -59 | -3 | 6.92 |
|  | Cluster2 | MOG.L | 1684 | -32 | -71 | 36 | 6.15 |
|  | Cluster3 | PoCG.R | 1028 | 38 | -33 | 59 | 6.03 |
|  | Cluster4 | FFG.L | 883 | -27 | -54 | -12 | 5.92 |
|  | Cluster5 | PCUN.R | 169 | 12 | -78 | 57 | 5.17 |
|  | Cluster6 | ITG.R | 209 | 50 | -63 | -5 | 4.90 |
|  | Cluster7 | MTG.L | 411 | -60 | -41 | -14 | 4.59 |
|  | Cluster8 | STG.L | 194 | -53 | -15 | 0 | 3.75 |
| SVCI-TUG | Cluster1 | THA.L | 772 | -8 | -18 | 6 | 4.16 |
| SVCI-Step length | Cluster1 | THA.R | 1352 | 20 | -14 | 9 | 4.75 |
| SVCI-Gait speed | Cluster1 | THA.L | 1458 | -12 | -14 | 17 | 6.00 |

GMV: gray matter volume, AD: Alzheimer's disease, SVCI: subcortical vascular cognitive impairment, DTUG: dual-task timed up and go test, DTC: dual-task cost, TUG: timed up and go test, LING.R: right lingual gyrus, HIP.R: right hippocampus, FFG.L: left fusiform gyrus, TPOsup.R: right temporal pole: superior temporal gyrus, MOG.L: left middle occipital gyrus, PoCG.R: right postcentral gyrus, PCUN.R: right precuneus, ITG.R: right inferior temporal gyrus, MTG.R: right middle temporal gyrus, STG.L: left superior temporal gyrus, THA.R: right thalamus, THA.L: left thalamus.

**Table *S6* Coordinates of brain regions in the positively and negatively CBF patterns for gait parameters in AD and SVCI groups**

| Group- gait metrics |  | Brain regions | Cluster size  (voxel) | MNI coordinate | | | *F* values |
| --- | --- | --- | --- | --- | --- | --- | --- |
|  |  |  |  | X | Y | Z |  |
| AD-DTUG | Cluster1 | PCUN.R | 917 | 14 | -60 | 34 | 5.67 |
| AD-BBS | Cluster1 | PCUN.L | 228 | -8 | -50 | 76 | 4.15 |
| AD-Gait speed | Cluster1 | IPL.L | 248 | -26 | -60 | 42 | 4.35 |
| SVCI-TUG | Cluster1 | CRBLCrus2.L | 581 | -26 | -74 | -38 | 6.04 |
| SVCI-DTUG | Cluster1 | CRBLCrus2.L | 362 | -26 | -74 | -40 | 4.86 |
| SVCI-BBS | Cluster1 | CRBLCrus2.L | 1213 | -18 | -82 | -42 | 7.10 |

CBF: cerebral blood flow, AD: Alzheimer's disease, DTUG: dual-task timed up and go test, SVCI: subcortical vascular cognitive impairment; TUG: timed up and go test, BBS: berg balance scale, PCUN.R: right precuneus, PCUN.L: left precuneus, IPL.L: left inferior parietal gyrus, CRBLCrus2.L: left cerebellum crus2.

**Table *S7* Coordinates of brain regions in the GMV for gait parameters in the AD-MCI, svMCI, ADD and sVD groups**

| Group-gait metrics |  | Brain regions | Cluster size (voxel) | MNI coordinate | | | *F* values |
| --- | --- | --- | --- | --- | --- | --- | --- |
|  |  |  |  | X | Y | Z |  |
| AD-MCI |  |  |  |  |  |  |  |
| DTC | Cluster 1 | TPOsup.R | 1569 | 47 | 11 | -18 | 6.35 |
|  | Cluster 2 | HIP.R | 681 | 27 | -24 | -8 | 3.84 |
| svMCI |  |  |  |  |  |  |  |
| Gait speed | Cluster 1 | IFGtri.L | 1061 | -33 | 27 | 2 | 5.39 |
| ADD |  |  |  |  |  |  |  |
| TUG | Cluster 1 | MTG.R | 794 | 54 | -72 | 0 | 6.82 |
|  | Cluster 2 | LING.L | 307 | -15 | -83 | -14 | 5.35 |
| DTUG | Cluster 1 | ITG.R | 1713 | 54 | -74 | -3 | 4.9 |
| Step length | Cluster 1 | CRBL9.L | 207 | -18 | -50 | -56 | 5.28 |
|  | Cluster 2 | SPG.L | 161 | -18 | -50 | -56 | 5.28 |
|  | Cluster 3 | PoCG.R | 594 | 43.5 | -33 | 56 | 6.04 |
| Gait speed | Cluster 1 | CRBL9.L | 229 | -18 | -50 | -56 | 6.00 |
| sVD |  |  |  |  |  |  |  |
| TUG | Cluster 1 | CRBL45.L | 1080 | -8 | -51 | -8 | 6.77 |

GMV: gray matter volume, AD-MCI: mild cognitive impairment due to probable Alzheimer’s disease, svMCI: subcortical vascular mild cognitive impairment, ADD: probable Alzheimer’s disease dementia; sVD: subcortical vascular dementia, TUG: timed up and go test, DTUG: dual-task timed up and go test, DTC: dual-task cost, TPOsup.R: right temporal pole: superior temporal gyrus, HIP.R: right hippocampus, IFGtri.L: left inferior frontal gyrus, triangular part, MTG.R: right middle temporal gyrus, LING.L: left lingual gyrus, ITG.R: right inferior temporal gyrus, PoCG.R: right postcentral gyrus, SPG.L: left superior parietal gyrus, CRBL9.L: left cerebellum9, CRBL45.L: left cerebellum 4_5.

**Table *S8* Coordinates of brain regions in the CBF patterns for gait parameters in the AD-MCI, svMCI, ADD and sVD group**

| Group-gait metrics |  | Brain regions | Cluster size (voxel) | MNI coordinate | | | *F*values |
| --- | --- | --- | --- | --- | --- | --- | --- |
|  |  |  |  | X | Y | Z |  |
| AD-MCI |  |  |  |  |  |  |  |
| DTC | Cluster 1 | PCG.L | 89 | -2 | -36 | 30 | 4.66 |
| svMCI |  |  |  |  |  |  |  |
| CV of gait speed | Cluster 1 | CRBLCrus2.L | 739 | -42 | -66 | -50 | 4.83 |
| ADD |  |  |  |  |  |  |  |
| Cadence | Cluster 1 | SPG.R | 616 | 20 | -46 | 70 | 7.81 |
| BBS | Cluster 1 | SMG.L | 166 | -62 | -28 | 24 | 4.32 |
|  | Cluster 2 | PoCG.R | 316 | 20 | -42 | 70 | 4.41 |
| sVD |  |  |  |  |  |  |  |
| BBS | Cluster 1 | CRBLCrus2.R | 307 | 4 | -90 | -34 | 4.98 |
|  | Cluster 2 | CRBL8.L | 483 | -22 | -52 | -50 | 4.62 |

CBF: cerebral blood flow, AD-MCI: mild cognitive impairment due to probable Alzheimer’s disease, svMCI: subcortical vascular mild cognitive impairment, ADD: probable Alzheimer’s disease dementia; sVD: subcortical vascular dementia, DTC: dual-task cost, BBS: berg balance scale, PCG.L: left posterior cingulate gyrus, SPG.R: right superior parietal gyrus, PoCG.R: right postcentral gyrus, SMG.L: left supramarginal gyrus, CRBLCrus2.R: right cerebellum crus2, CRBLCrus2.L: left cerebellum crus2, CRBL8.L: left cerebellum8.

Table *S9* Coordinates of brain regions in the difference of GMV and CBF in AD-MCI, svMCI and HC groups

| Group |  | Brain regions | Cluster size (voxel) | MNI coordinate | | | F values |
| --- | --- | --- | --- | --- | --- | --- | --- |
|  |  |  |  | X | Y | Z |  |
| GMV |  |  |  |  |  |  |  |
| HC>AD-MCI | Cluster 1 | HIP.R | 921 | 22.5 | -15 | -16.5 | 4.36 |
| HC>svMCI | Cluster 1 | THA.R | 6517 | 14 | -17 | 0 | 5.09 |
|  | Cluster 2 | PoCG.R | 3407 | 45 | -20 | 45 | 4.88 |
|  | Cluster 3 | PoCG.L | 2008 | -47 | -20 | 38 | 4.78 |
| CBF |  |  |  |  |  |  |  |
| HC>AD-MCI | Cluster 1 | ITG.L | 663 | -62 | -56 | -8 | 4.4 |
| HC>svMCI | Cluster 1 | MTG.R | 922 | 60 | -48 | 2 | 4.33 |

GMV: gray matter volume, CBF: cerebral blood flow, AD-MCI: mild cognitive impairment due to probable Alzheimer’s disease, svMCI: subcortical vascular mild cognitive impairment, HC: healthy controls, CBF: cerebral blood flow, HIP.R: right hippocampus, THA.R: right thalamus, PoCG.R: right postcentral gyrus, PoCG.L: left postcentral gyrus, ITG.L: left inferior temporal gyrus, MTG.R: right middle temporal gyrus

**
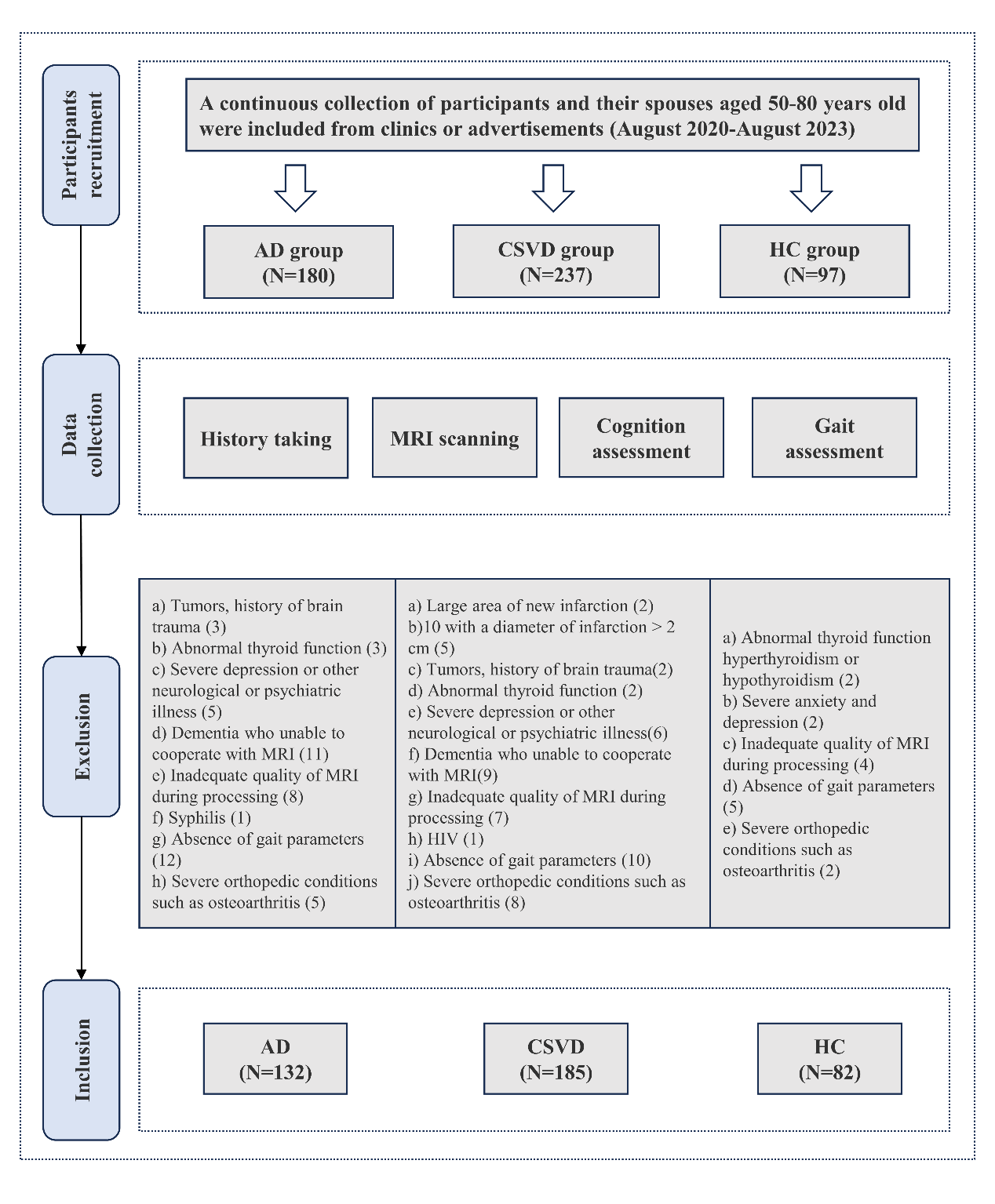
**

**Fig. *S1* Flow chart of inclusion and exclusion criteria**

AD: Alzheimer’s disease, CSVD: cerebral small vessel disease, HC: healthy controls

**
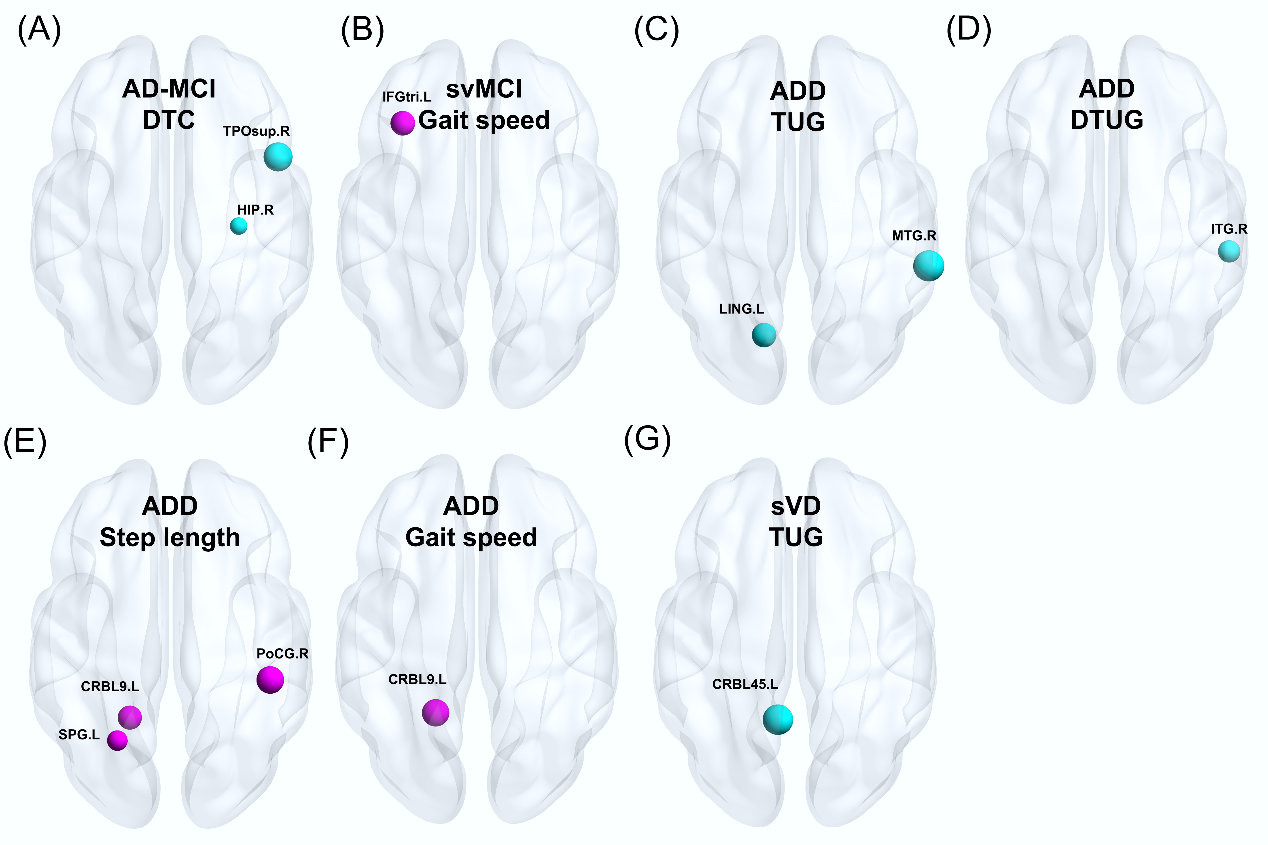
**

**Fig. *S2* Significant clusters comprising the GMV patterns for gait parameters in the AD-MCI, svMCI, ADD and sVD groups**

(FWE correction, *P* < 0.0125; corrected for age, gender, BMI, and TIV). GMV: gray matter volume, AD-MCI: mild cognitive impairment due to probable Alzheimer’s disease, svMCI: subcortical vascular mild cognitive impairment, ADD: probable Alzheimer’s disease dementia, sVD: subcortical vascular dementia, BMI: body mass index, TIV: total intracranial volume, TUG: timed up and go test, DTUG: dual-task timed up and go test, DTC: dual-task cost, TPOsup.R: right temporal pole: superior temporal gyrus, HIP.R: right hippocampus, IFGtri.L: left inferior frontal gyrus, triangular part, MTG.R: right middle temporal gyrus, LING.L: left lingual gyrus, ITG.R: right inferior temporal gyrus, PoCG.R: right postcentral gyrus, SPG.L: left superior parietal gyrus, CRBL9.L: left cerebellum9, CRBL45.L: left cerebellum 4_5.

**
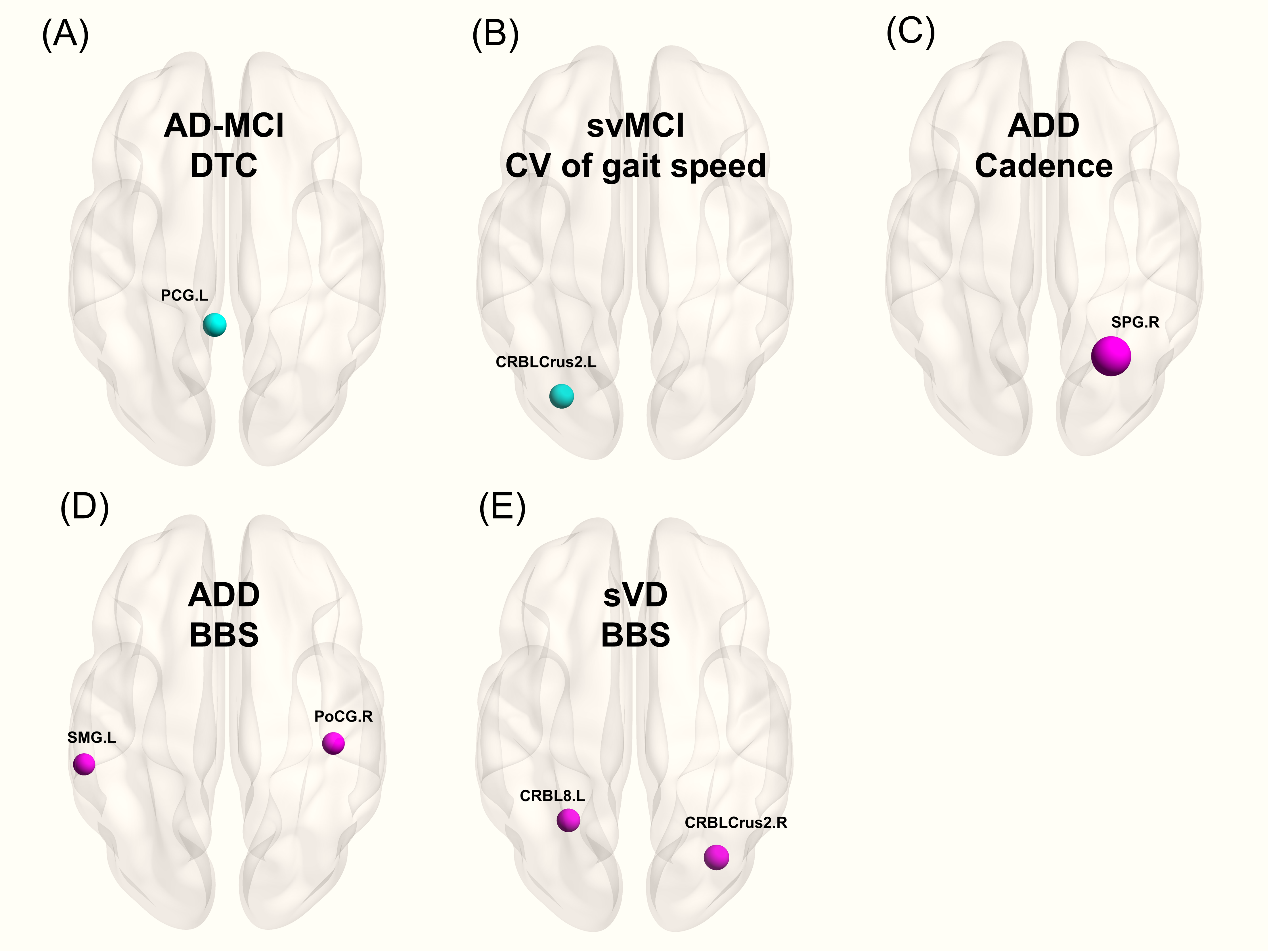
**

**Fig. *S3* Significant clusters comprising the CBF patterns for gait parameters in the AD-MCI, svMCI, ADD and sVD groups**

(FWE correction, *P* < 0.0125; corrected for age, gender, and BMI). CBF: cerebral blood flow, AD-MCI: mild cognitive impairment due to probable Alzheimer’s disease, svMCI: subcortical vascular mild cognitive impairment, ADD: probable Alzheimer’s disease dementia, sVD: subcortical vascular dementia, BMI: body mass index, DTC: dual-task cost, BBS: berg balance scale, PCG.L: left posterior cingulate gyrus, SPG.R: right superior parietal gyrus, PoCG.R: right postcentral gyrus, SMG.L: left supramarginal gyrus, CRBLCrus2.R: right cerebellum crus2, CRBL8.L: left cerebellum8, CRBLCrus2.L: left cerebellum crus2.

**References**

1. Lu J, Li D, Li F, Zhou A, Wang F, Zuo X, et al. Montreal cognitive assessment in detecting cognitive impairment in chinese elderly individuals: A population-based study. J Geriatr Psychiatry Neurol. Dec 2011;24(4):184-90.

2. Gmitrowicz A, Kucharska A. [developmental disorders in the fourth edition of the american classification: Diagnostic and statistical manual of mental disorders (dsm iv -- optional book)]. Psychiatr Pol. Sep-Oct 1994;28(5):509-21. Zaburzenia psychiczne wieku rozwojowego w roboczej wersji IV edycji klasyfikacji amerykańskiej--Diagnostic and Statistical Manual of Mental Disorders (DSM IV--Options Book).

3. Fazekas F, Kleinert R, Offenbacher H, Schmidt R, Kleinert G, Payer F, et al. Pathologic correlates of incidental mri white matter signal hyperintensities. Neurology. Sep 1993;43(9):1683-9.

4. Staals J, Makin SD, Doubal FN, Dennis MS, Wardlaw JM. Stroke subtype, vascular risk factors, and total mri brain small-vessel disease burden. Neurology. Sep 30 2014;83(14):1228-34.

5. Xu G, Rowley HA, Wu G, Alsop DC, Shankaranarayanan A, Dowling M, et al. Reliability and precision of pseudo-continuous arterial spin labeling perfusion mri on 3.0 t and comparison with 15o-water pet in elderly subjects at risk for alzheimer's disease. NMR Biomed. Apr 2010;23(3):286-93.

6. Eklund A, Nichols TE, Knutsson H. Cluster failure: Why fmri inferences for spatial extent have inflated false-positive rates. Proc Natl Acad Sci U S A. Jul 12 2016;113(28):7900-5.

7. Yan CG, Wang XD, Zuo XN, Zang YF. Dpabi: Data processing & analysis for (resting-state) brain imaging. Neuroinformatics. Jul 2016;14(3):339-51.
